# On the Design of Steep Optical Absorbers for Vacuum‐Processed Organic Solar Cells: One Isopropyl Group Makes the Difference

**DOI:** 10.1002/smsc.202500633

**Published:** 2026-05-15

**Authors:** Mohamed el habib Bouajhine, Emilio Lorini, Samuele Giannini, Siebe Frederix, Karsten Walzer, Marieta Levichkova, Gunter Mattersteig, Martin Pfeiffer, Eva Bittrich, Petra Uhlmann, Vincent Lemaur, Koen Vandewal, Patrick Brocorens, Luca Muccioli, David Beljonne

**Affiliations:** ^1^ Laboratory for Chemistry of Novel Materials University of Mons Mons Belgium; ^2^ Department of Industrial Chemistry University of Bologna Bologna Italy; ^3^ Department of Chemistry and Industrial Chemistry University of Pisa Pisa Italy; ^4^ imec, Institute for Materials Research (imo‐imomec) Hasselt University Diepenbeek Belgium; ^5^ imo‐imomec Energyville Genk Belgium; ^6^ Heliatek GmbH Dresden Germany; ^7^ Leibniz‐Institut für Polymerforschung Dresden e.V. Dresden Germany

**Keywords:** exciton–vibronic interactions, molecular packing, steep optical absorption, structure–property relationships, vacuum‐deposited organic solar cells

## Abstract

The design of vacuum‐processed organic solar cells requires a balance between optimizing energetic and morphological properties for efficient photovoltaic performance. In this study, we investigate two structurally similar molecular donors, DCV‐iPr and DCV‐Me, which exhibit stark differences in photovoltaic performance despite their minimal chemical differences. Through a combined experimental and theoretical approach—including crystal structure prediction, vapor deposition simulations, GIWAXS measurements, and advanced electronic structure calculations—we establish how molecular packing influences optical absorption and photovoltaic efficiency. Our findings show that steric effects introduced by the isopropyl group in DCV‐iPr lead to a brick‐wall molecular arrangement, favoring J‐like excitonic interactions and resulting in sharp optical absorption with a reduced Stokes shift. In contrast, DCV‐Me forms a more H‐like aggregation, leading to broadened absorption and higher voltage losses. While DCV‐iPr demonstrates enhanced photovoltaic performance, we identify substantial remaining voltage losses associated with charge–transfer excitations at the donor–acceptor interface. This work provides key design guidelines linking molecular packing to optical absorption properties and highlights the need for alternative acceptor materials with steeper absorption onsets to further optimize vacuum‐processed organic solar cells.

## Graphical abstract

A minor steric modification in molecular donors (DCV‐iPr vs DCV‐Me) leads to distinct packing motifs, which govern excitonic interactions and optical properties in vacuum‐processed organic solar cells. DCV‐iPr forms a brick‐wall, J‐like aggregated structure with sharp absorption and improved photovoltaic performance, whereas DCV‐Me exhibits H‐like aggregation with broader absorption and higher voltage losses. Despite improved efficiency, interfacial charge‐transfer losses remain significant, highlighting the need for acceptors with sharper absorption onsets

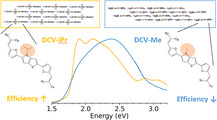

## 1 Introduction

The development of clean, renewable energy sources is essential to mitigate climate change. Solar power offers an unlimited supply of energy, provided its efficient harnessing. Next to silicon solar cells, whose applications are limited by their high weight of >20 kg/m^2^, organic photovoltaics (OPV) has the potential for large‐scale production of lightweight (<2 kg/m^2^) and flexible modules for utilization at vast façade and roof areas [1]. It further corresponds to the “greenest” and most responsible of all renewable energy technologies due to consequent use of non‐toxic, earth‐abundant elements, and an ultra‐short energy payback time [2, 3].

Recently, OPV has seen a drastic increase in power conversion efficiency (PCE), with solution‐processed devices achieving 20.1% at lab conditions [4, 5, 6, 7, 8]. However, these record performances were obtained with molecules of high synthetic complexity, not scalable to production [9]. Their efficiency benefit often gets lost upon upscaling to areas >100 cm^2^. Also, wet processing often requires the use of potentially harmful solvents and is critical for achieving industry‐relevant module lifetime. In contrast, the possibility to scale‐up small‐molecule OPV materials for vacuum processing to multi‐kilogram scale, combined with marginal efficiency losses when scaling to large areas, offers an environmentally friendly route to OPV. As the first OPV product worldwide, 2024 Heliatek's vacuum‐deposited OPV modules achieved all efficiency and lifetime test cycles demanded for IEC61215:2021 certification [10]. It is noteworthy to point out that all‐evaporated organic photovoltaic devices, as prepared in Ref. [11, 12, 13], reached certified efficiencies exceeding 10%.

Nevertheless, further progress is needed: both solution and vacuum‐based OPV suffer from non‐radiative energy losses limiting their open‐circuit voltage (V_oc_), which is considered the main obstacle for further improvement of the PCE and the reason why efficiencies of OPV are still lower than of other PV technologies [14, 15]. While concise material design paths to achieve predictable V_oc_ reduction are not yet known [16], it is clear that molecular packing plays an important role. Besides being essential for achieving good charge transport properties, a proper spatial arrangement of the molecular building blocks in the solid thin films is needed to minimize energetic disorder, which results in sharp absorption onsets. In particular, it is well‐established that molecules adopting H‐like (face‐to‐face) molecular stacking feature broadened absorption bands [17, 18], which results in increased losses associated with energetic disorder. In contrast, a slipped stacking arrangement enables J‐like excitonic coupling and leads to narrow and red‐shifted absorption bands [19], translating into reduced energetic disorder and voltage losses. J‐like aggregation is therefore expected to promote spectral steepness, which, everything else remaining the same, should turn into improved OPV efficiency. All this, together with an appropriate energetic alignment, is required to achieve low voltage losses, as it would bring the effective gap of the organic semiconductor blends close to the energies at which photons are strongly absorbed. The effective gap of an organic photovoltaic blend is the energy of the charge–transfer state (E_CT_) formed between electron donating and accepting molecules. In the best organic solar cells today, E_CT_ approaches the singlet energy of the neat materials. At these low driving forces, it has been shown that steep absorption onsets are required to maintain a high yield of exciton dissociation [20].

The steepness of an absorption spectrum has two aspects. First, one can use the slope of a normalized linear plot of the optical density at the inflection point of the absorption edge as a measure for the steepness. This quantity, referred to as steepness of the absorption edge in this manuscript, is meaningful for solar cell performance, since a low steepness leads to a wider region in the external quantum efficiency (EQE) spectrum with suboptimum EQE values next to the absorption edge and thus to suboptimum short circuit current (J_sc_) for a material with given absorption onset. Moreover, a high steepness of the absorption edge is a necessary condition for a high overlap integral between absorption and photoluminescence and is thus crucial for efficient exciton diffusion by Förster transfer. Even though the absorption edge steepness may also be affected by disorder (inhomogeneous broadening), we will elaborate below that it is primarily determined by the complex interplay between J‐ and H‐type interactions in organic materials, which can lead to very different steepness even in absence of any disorder. For a nanocrystalline material, we expect that the shape of the absorption edge is governed by the dominant aggregation type, while nanocrystallite size and crystal imperfection will also play a role.

On the other hand, the presence of both dynamic (electron–phonon coupling) and static disorder (structural or conformational) will lead to a tail in the absorption spectrum related to subgap states. This steepness of the absorption tail is commonly defined as the inverse of the Urbach energy, and can be derived as the logarithmic slope dln (α)/dE of the exponential absorption tail [21]. Experimentally, an Urbach‐type slope can be extracted from sensitive EQE measurements by fitting the linear region of ln (EQE) versus photon energy in the sub‐gap regime. However, in photovoltaic donor–acceptor blends, the sub‐gap absorption is frequently dominated by CT states and may additionally be influenced by mid‐gap trap states. In such cases, the extracted quantity represents an apparent Urbach energy rather than the intrinsic band‐edge disorder of the neat material. Nevertheless, this apparent steepness is widely used as a metric to compare sub‐gap spectral broadening across different material systems [22, 23]. To get a characterization of the influence of disorder, it is more meaningful to evaluate the slope of the EQE in the region below the CT shoulder.

**FIGURE 1:**
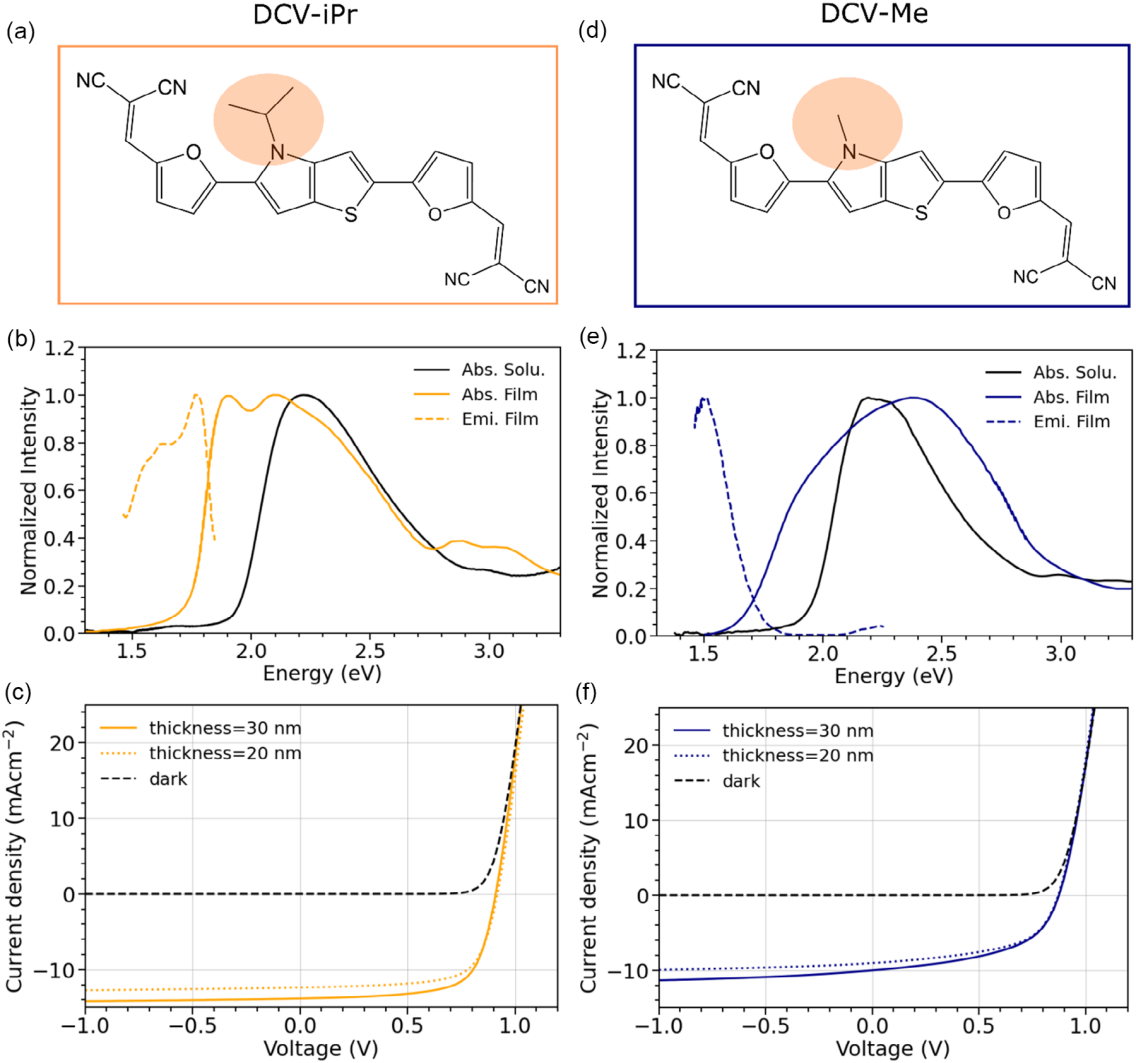
Panels (a) and (d) represent the chemical structures of DCV‐iPr and DCV‐Me, respectively, highlighting the difference in the N‐alkyl moiety. Panels (b) and (e) report the experimental spectra of DCV‐iPr and DCV‐Me, respectively. The spectra recorded in dimethylacetamide (DMAc) solution are represented with solid black lines, while the absorption and photoluminescence in the 30 nm thick thin film are represented with solid and dashed lines, respectively. Panels (c) and (f) report the J–V curves of DCV‐iPr and DCV‐Me bulk heterojunction (BHJ) solar cells, where the molecules act as donor in a mixture with fullerene C_60_ as acceptor, respectively. The colored lines represent the J–V characteristics of the BHJ with 20 nm (dashed lines) and 30 nm (solid lines) absorber thickness, respectively. The black lines are the dark current characteristics of the same devices.

In order to shed light on the factors that determine the molecular packing in vacuum deposited OPV and its relation with a sharp absorption onset, here we investigate two closely related molecules, based on furane‐dicyanovinylene end‐capped fuzed aromatic rings: DCV‐Fu‐TPy‐Fu‐iPr(3) and DCV‐Fu‐TPy‐Fu‐Me(3), referred to hereafter as DCV‐iPr and DCV‐Me, respectively. Even though they differ only by the substitution of one methyl side group by an isopropyl (see Figure 1a,d), they yield dramatically different steady state spectra in their thin‐film phases as well as photovoltaic response when used as donors in either planar or bulk heterojunction cells utilizing C_60_ as acceptor. As discussed below, the poorly performing DCV‐Me molecule shows a broad, featureless, optical absorption spectrum with an absorption edge steepness of only 2.4 eV^−1^, and a large Stokes shift in its pure thin film phase (see Figure 1b,e). The better performing DCV‐iPr donor instead displays a sharp low‐energy absorption edge with a steepness of 9.8 eV^−1^, a well‐defined vibronic fine structure and a reduced Stokes shift. In Figure S23, the absorption spectra of DCV‐iPr and DCV‐Me are plotted together with a state‐of‐the‐art A‐D‐A‐type small molecule absorber used for vacuum deposited OPV, a methyl‐substituted dicyanovinyl (DCV)‐capped A−D−A quinquethiophenes (DCV5T‐Me) [24], which has an absorption edge steepness of 4.8 eV^−1^. It is remarkable that DCV‐iPr and DCV‐Me, respectively, have either much higher or much lower steepness than DCV5T‐Me, depending only on their alkyl substituent. By combining GIWAXS measurements and crystal structure predictions (CSP), we demonstrate that the differences in optical spectra are related to changes in molecular packing. Thin film structures were simulated with a well‐established computational method [25, 26, 27], replicating the results of experimental vapor deposition. Using such realistic structures accounting for structural disorder in thin films, we calculate the optical absorption and emission spectra using an advanced Frenkel–Holstein model and reproduce the salient features of the corresponding experimental data. Most importantly, we find that steric effects associated with the presence of the bulky isopropyl groups entail a slipped‐stacked configuration of the molecular building blocks, favoring J‐like interactions and a sharp absorption edge.

A detailed analysis of the voltage losses in the better‐performing DCV‐iPr‐based solar cells further shows that, in addition to the steepness of the absorption edge and tail, it is equally important to tune the energy levels of the donor and acceptor molecules in close resonance in order to minimize losses associated with the formation of low‐lying charge–transfer excitations. Finally, we show efficiency‐optimized photovoltaic devices reaching up to 9.5% based on a combination of DCV‐iPr:C_60_ blend and a pristine layer of BODIPY‐based near‐infrared (NIR) absorber.

## 2 Results and Discussion

### 2.1 Photovoltaic Response

Photovoltaic devices were fabricated by vacuum deposition in a typical n‐i‐p device architecture consisting of glass ITO/C_60_ (15 nm)/Donor:C_60_/HTL (10 nm)/HTL:NDP9 (30 nm)/NDP9 (1 nm)/Al (100 nm). The 15 nm of neat C_60_ acts as an electron extraction layer to the transparent indium tin oxide (ITO) electrode. To extract the holes, a layer stack of neat hole transport layer (HTL) material, followed by p‐doped HTL with 10% of NDP9 as dopant and 1 nm of pure NDP9, is used (see Section 4.1 for further details).

Figure 1c,f, show optimized devices for both donor materials, with the active layer thickness of 20 and 30 nm, respectively. Section S1 in Supporting Information shows the corresponding EQE of the best cell with DCV‐iPr as well as the dependence of FF and V_oc_ on the irradiance for DCV‐iPr and DCV‐Me devices. For both donors, the optimum mixing ratio and substrate temperature were determined. The best performing devices with DCV‐iPr were achieved in a 2:1 mass ratio at 50°C substrate temperature, while best devices of DCV‐Me required a mixing ratio of 1:1 at ambient temperature. The DCV‐iPr:C_60_ solar cell reaches a power conversion efficiency (PCE) of 7.6% after spectral mismatch correction (MMC). This is a respectable efficiency for a fully vacuum deposited organic solar cell, in this case optimized to harvest photons with wavelengths below 700 nm and intended for use as a high gap, high voltage top cell in fully vacuum deposited tandem or triple‐stack OPV. In contrast, DCV‐Me:C_60_ was found to have a drastically reduced PCE of 4.6%, due to a reduction in all photovoltaic parameters: V_oc_ drops from 0.91 V to 0.88 V, FF from 0.69 to 0.52, and J_sc_ from 12.04 mA/cm^2^ (after MMC) to 10.0 mA/cm^2^. This unexpectedly large difference in photovoltaic response for such a small difference in chemical structure triggered further investigation into the microstructural factors at its origin.

### 2.2 Experimental Optical Absorption and Photoluminescence Spectra

The measured steady‐state spectra of DCV‐iPr and DCV‐Me in dimethylacetamide (DMAc) solution and in their thin‐film phases are shown in Figure 1b,d. The samples were prepared as described in Section 4.1. As expected for molecules with the same chemical core, their absorption spectra in dilute DMAc solution are very similar, with a main absorption peak at about $E_{S_{0}\to S_{1}}^{\left(\mathrm{Sol}\right)}$= 2.19 eV (see also Figure S9). Time‐dependent density functional theory (TDDFT) calculations performed on both systems (as detailed in Section S3) reveal that the two molecules have essentially the same electronic transition (with a predominant HOMO–LUMO character) localized on the molecular core. Notably, the absorption spectra of both molecules in solutions are equally broad with a skewed tail at higher energies (see Figure 1b,e), which is indicative of a vibronic progression concealed by the broadening, owing to the nuclear dynamics of the molecule and to the solvent reorganization. An analog shoulder is also present in the photoluminescence spectrum of DCV‐iPr in DMAc solution (see Figure S9).

To confirm the origin and the nature of the observed features, a TDDFT analysis at ωB97X‐D/6‐31G(d, p) level, combined with a quantum displaced harmonic oscillator model [19, 28, 29] was performed for both DCV‐iPr and DCV‐Me. As explained in Section S4–6, this analysis reveals the presence of vibrational modes strongly coupled with the excitation in the region above 1100 cm^−1^. Among these high‐frequency modes, those in the region of 1500 and 1600 cm^−1^, which are the ubiquitous stretching/aromatic ring‐breathing modes, have sizable weights on the backbone unit of the molecule and are largely contributing to the total exciton relaxation energy, $\lambda^{\mathrm{rel}}$ (see Figure S8). As commonly done in the literature [17, 30, 31], such high‐frequency modes with large exciton—phonon coupling can be conveniently coalesced in a single effective vibrational mode, which in this case has a frequency, $\hbar \omega_{\mathrm{eff}}=1532$ cm^−1^ and an effective Huang Rhys factor $\left(S_{\mathrm{eff}}=\lambda_{\mathrm{hf}}^{\mathrm{rel}}/\hbar \omega_{\mathrm{eff}}\right)$ of 0.646. Details on these calculations can be found in Section S4. Note that, along with high‐frequency modes, there are several low‐frequency modes essentially related to torsional degrees of freedom between the central donor and the acceptor side groups. These modes contributed to the homogeneous broadening of the spectrum with a standard deviation $\sigma^{\mathrm{hom}.}∼46$ meV.

Using the $\omega_{\mathrm{eff}}$ and $S_{\mathrm{eff}}$ and the homogeneous broadening to calculate the molecular absorption (Equation S4), we can reproduce the vibronic progression of the main $S_{0}\;\to \;S_{1}$ transition in the DCV‐derivatives. Our calculations also replicate the Stokes shift between the absorption and emission spectra in dilute solution, confirming the quality of the functional used to describe the vibronic properties of the DCV‐derivatives investigated (see simulated absorption and emission spectra in Figure S9). We note, however, that TDDFT overestimates the excitation energy with respect to the main experimental band by ∼0.5 eV in vacuo, and by ∼0.4 eV when accounting for the environment with a polarizable continuum model [32]. Since we are primarily interested in understanding spectral changes upon aggregation, the conclusions of our analysis below are unaffected by this discrepancy.

When going from solution to the thin‐film solid‐state phase, both systems show significant changes in their experimental absorption spectra (see Figure 1b,e). As anticipated, although the two molecules are chemically very similar and show comparable features in solution, the solid‐state spectra of DCV‐iPr and DCV‐Me are remarkably different. Namely, the DCV‐iPr thin‐film absorption spectrum retains a structured profile in the lower energy region, remains sharp, and exhibits a maximum red‐shifted of about 0.27 eV compared to that in solution. The red shift is accompanied by the appearance of a second peak at 2.08 eV, which is ∼0.19 eV higher than the first, attributed to the 0–0 transition. The second peak is reminiscent of the vibronic shoulder already observed in solution, but with an enhanced intensity. In contrast, the DCV‐Me solid‐state spectrum becomes featureless, and much broader compared to that of DCV‐iPr, with a tail at higher energies and a ∼0.2 eV blueshifted maximum compared to the solution. Similar striking differences are also visible in the photoluminescence (PL) spectra of the thin films of DCV‐iPr and DCV‐Me. We observe a large Stokes shift for DCV‐Me emission, while DCV‐iPr shows a smaller Stokes shift of ≈0.12 eV. As for the absorption onset, the PL of DCV‐iPr displays a sharp peak at 1.77 eV together with a vibronic shoulder at 1.59 eV. In contrast, DCV‐Me exhibits a less steep emission with a peak at about 1.51 eV.

In summary, from the analysis of the UV/VIS spectra, many questions arise, that we will address in the following: what causes the changes in the optical spectra of DCV‐Me and DCV‐iPr when transitioning from solution to the solid state? Why do the two molecules, despite their chemical similarity, exhibit significantly different responses in the solid state? Do these spectral changes influence the photovoltaic performance of the respective devices?

### 2.3 Single Crystal and Thin Film Phase Structures

While the attempts to grow single crystals of DCV‐iPr were successful, this was not the case for DCV‐Me, which could be synthesized in small amounts only. Furthermore, polymorphism in molecular materials—especially in thin films—is more the rule than the exception, meaning the obtained single crystal structure may not necessarily represent the films measured by optical spectroscopy. We thus conducted an in‐depth theoretical structural analysis built on two key pillars: (i) identification of the most stable polymorphs for the 3D bulk single crystals of the two molecules, and (ii) simulations of layer‐by‐layer growth of the corresponding thin films. Finally, the consistency between the predicted single crystal and thin‐film structures was assessed by comparing the calculated and measured 1D and 2D GIWAX patterns of 30 nm DCV‐iPr and DCV‐Me thin films on C_60_.

#### 2.3.1 Single Crystal Structures

DCV‐iPr and DCV‐Me polymorphs were obtained using two independent crystal structure prediction (CSP) approaches: one relying on DFT optimization of bulk phase polymorphs generated using simulated annealing (bulk CSP, BCSP) and another employing solely classical MD simulations in the presence of a flat surface (surface CSP, SCSP). A conformational search on the isolated molecules led to two different conformations, referred to as C‐shape and S‐shape (see also Section S2). While the C‐shape molecule is more stable at the molecular level, the lowest energy crystal structures are instead those built using the S‐shape conformer, highlighting the subtle interplay between intra‐ and inter‐molecular interactions in driving the energetics of crystal formation (see Section S7). The most stable polymorph obtained for DCV‐iPr from BCSP simulations is described by the P2_1_ space group, i.e., it is a monoclinic crystal with two molecules per cell (that turns out to match very well the measured single crystal, see below). In view of the important changes in energy ranking of DCV‐iPr conformations when going from isolated molecules to solid state, we applied the bulk CSP method on all the possible conformations of the isolated DCV‐Me molecule identified through conformational search, i.e., 16 in total (see Table S2). The polymorph search led to a proposed P2_1_/c crystal with a conformation that is ranked 3rd as an isolated molecule. Like DCV‐iPr, the crystalline cell of DCV‐Me is monoclinic, but it contains four molecules per unit cell instead of two (see Table 1).

**TABLE 1:** Lattice lengths (Å), angles (°), formula units (Z) of the crystalline cells obtained through BCSP, and SCSP for both molecules.

		a	b	c	α	β	γ	Z
DCV‐iPr	Exp	8.7	6.8	17.5	90.0	90.8	90.0	2
	BCSP	8.7	6.9	17.5	90.0	90.2	90.0	2
	SCSP	8.7	6.9	17.5	89.1	88.5	88.7	2
DCV‐Me	BCSP	7.5	8.6	29.4	90.0	94.8	90.0	4
	SCSP	8.2	8.5	27.8	90.6	98.5	90.4	4

The surface CSP method was employed, focusing on the S‐shaped conformation of both molecules. Polymorphs analogous to the ones obtained from bulk CSP were found for both molecules, albeit showing slightly different parameters associated with surface effects. As a matter of fact, optimizing at the same level of theory (DFT‐D with PBE) these thin‐film structures in bulk conditions, imposing 3D periodic boundary conditions, exactly restores the lattice parameters of the single crystals (see Section S7, Table S6 and S9). Table 1 collects the single crystal structural parameters of the most stable polymorph for the two molecules using either BCSP simulations or SCSP upon bulk re‐optimization. In the case of DCV‐iPr, as alluded to above, single crystals were grown in vacuum thermal gradient sublimation as described below in Methods. The corresponding lattice parameters, also listed in Table 1, match remarkably well the predicted BCSP and SCSP results.

#### 2.3.2 Diffraction Powder Pattern and GIWAXS

**FIGURE 2:**
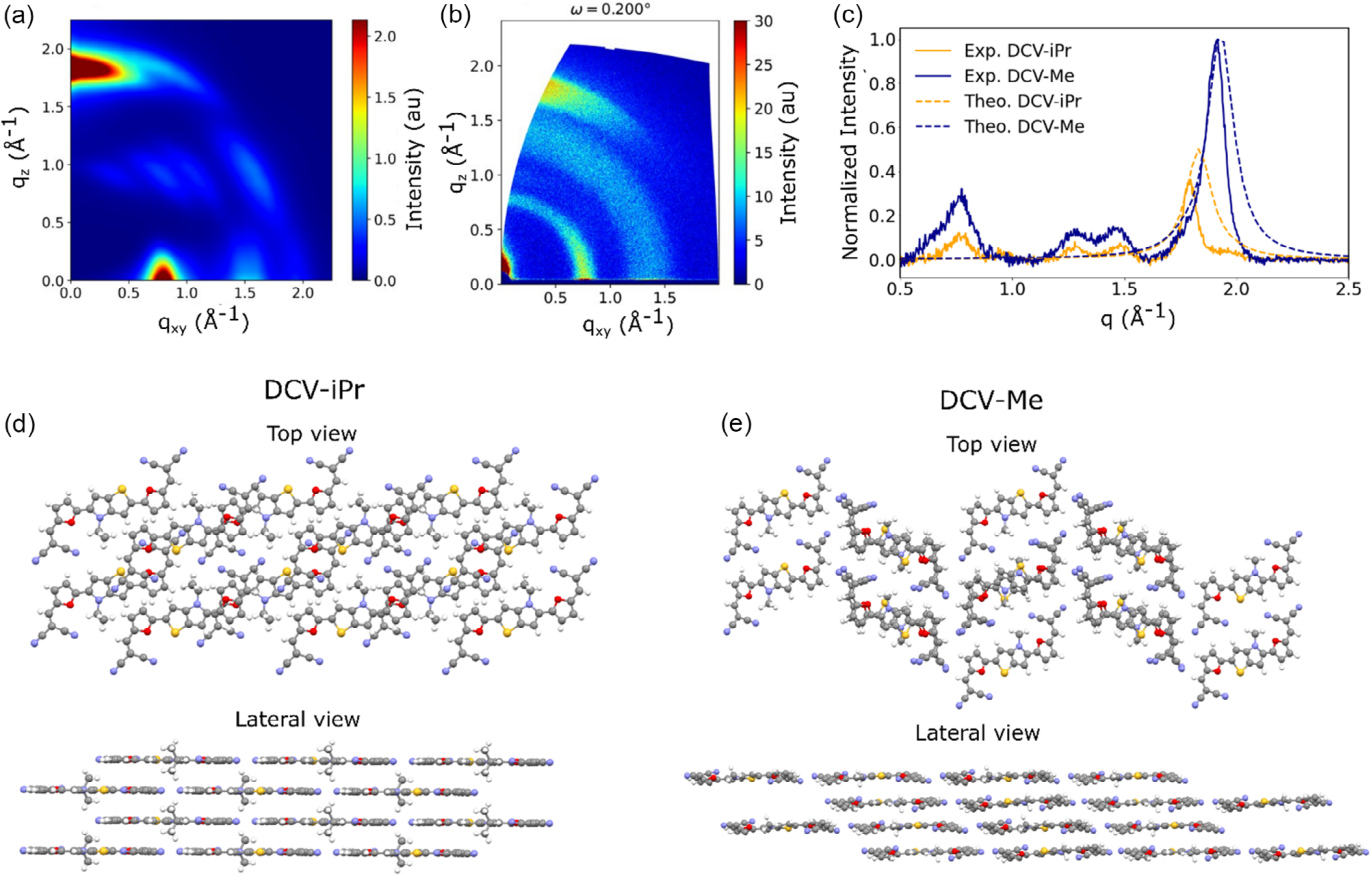
(a) Simulated 2D GIWAXS diagram of DCV‐iPr. (b) Experimental GIWAXS pattern of DCV‐iPr, including signals from the C_60_ sublayer. (c) Simulated (dashed lines) and experimental (continuous lines) 1D GIWAXS diagram of DCV‐Me (blue) and DCV‐iPr (orange). Measured GIWAXS diagrams on films including signals from the C_60_ sublayer; the blue and orange intense peaks represent DCV‐Me and DCV‐iPr, respectively, the three remaining peaks at low angles correspond to the fullerene sublayer. (d) Representation of the experimental DCV‐iPr crystal structure viewed from above and from the side. (e) Representation of simulated DCV‐Me crystal structure (using BCSP, see the text for details).

Powder diffractograms of DCV‐iPr were calculated for the predicted crystal structure (see Figure S10). They show two intense peaks corresponding to (100) and (020) diffractions at *q* = 0.8 and 1.83 ${\text{Å}}^{-1}$, respectively, which will be used to interpret the experimental 1D and 2D GIWAXS patterns, recorded to determine the orientation of the crystallites in thin films. A 2D GIWAXS pattern of a 30 nm DCV‐iPr layer deposited on a 10 nm fullerene layer (C_60_) is displayed in Figure 2b. Isotropic signals of the C_60_ sublayer can be found at *q* = 0.78 ${\text{Å}}^{-1}$ and 1.37 ${\text{Å}}^{-1}$ due to the choice of grazing incident angle. The 1D GIWAXS pattern at the same incident angle (Figure 2c) also features a fullerene signal with three peaks at *q* = 0.78 ${\text{Å}}^{-1}$, 1.28 ${\text{Å}}^{-1}$, and 1.47 ${\text{Å}}^{-1}$ (for more details see Methods). Most importantly, in the 2D GIWAXS pattern, we can identify two main spots attributed to DCV‐iPr and corresponding to the two most intense peaks in the powder diffractogram. The first spot appears close to the out‐of‐plane *q*_z_ axis. This peak is also clearly visible in the 1D GIWAXS pattern, which is an additional measurement of the out‐of‐plane direction. A spot at *q*_z_ = 1.83 Å^−1^ arises from the reflection of crystal planes parallel to the substrate on which the film is deposited, in this case (020) planes, and the corresponding interplanar distance of 3.43  Å corresponds to π–π interactions. Since the signal appears along the *q*_z_‐axis, we conclude that the orientation of the molecules relative to the substrate is preferentially “face‐on” (Figure S11).

The second intense spot in the 2D GIWAXS pattern of DCV‐iPr appears at a value $q_{xy}$ of 0.8 ${\text{Å}}^{-1}$, which corresponds to (100) planes, with an interplanar distance of 7.85 Å. As this spot appears mostly in‐plane in the GIWAXS pattern, it follows that (100) planes (represented by the black planes on Figure S11 in Supporting Information) are perpendicular to the substrate and represent lamellar thickness. To validate our interpretation, we simulated the corresponding GIWAXS patterns (2D and 1D), as described in Ref. [33], and found a good fit between the theoretical and experimental patterns (see Figure 2a–c). This agreement confirms the predictive power of the CSP tools used and, equally importantly, supports the view that the pure thin‐film phase of DCV‐iPr predominantly consists of the S‐shaped P2_1_ polymorph. Applying the Scherrer formula to the measured 1D GIWAXS data of the π−π stacking reflection leads to a lower limit of the out‐of‐plane coherence length of ∼7 nm (neglecting instrumental and geometrical broadening).

In the case of DCV‐Me, we can only rely on the measured GIWAXS patterns of the thin films, since as mentioned before, we were not able to isolate single crystals. The simulated powder pattern of the structure obtained by the CSP method is characterized by intense peaks at 0.82 ${\text{Å}}^{-1}$, 0.89 ${\text{Å}}^{-1}$, 1.89 ${\text{Å}}^{-1}$ and 1.91 ${\text{Å}}^{-1}$, corresponding to (011), (012), (211), and (204) planes, respectively (see Figure S10 in Supporting Information). The measured 1D GIWAXS experimental pattern in Figure 2c also shows an intense peak at ($q_{z}$=1.9 ${\text{Å}}^{-1}$, hence we conclude that the (204) plane lies essentially parallel to the substrate. Considering such an orientation, the simulated 1D GIWAXS pattern (see Figure 2c) agrees well with the experimental one. As the (204) planes are almost parallel to the molecular planes, a face‐on molecular orientation with respect to the substrate can be considered also for DCV‐Me. On the other hand, the $q_{z}$ value shows a spacing between planes of 3.29 Å, which is smaller than the corresponding value in DCV‐iPr, explaining the displacement towards larger *q*‐values of the corresponding peak of DCV‐Me compared to DCV‐iPr in both experimental and theoretical patterns. The lower limit of the out‐of‐plane coherence length was estimated as ∼6 nm, comparable to the one of DCV‐iPr films. At this stage, the following picture emerges regarding the structural organization of the molecules in the two films. While in DCV‐iPr, the presence of the bulky isopropyl groups leads to a longitudinal shift of the molecules with respect to one another and the formation of a 2D brickwall‐like arrangement, the smaller methyl groups in DCV‐Me afford stronger π−π interactions and closer intermolecular separation but with weaker communication between the quasi‐1D columns, see Figure 2d,e.

#### 2.3.3 Simulation of Vapor Deposition

**FIGURE 3:**
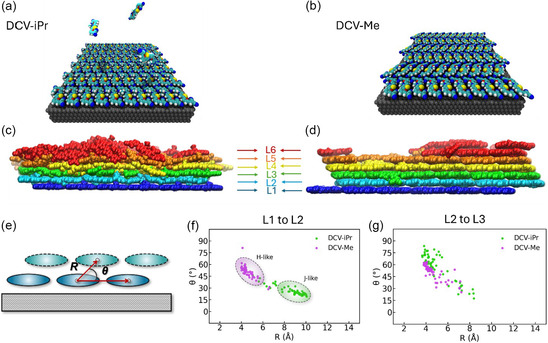
Panels (a) and (b): visualization of the template monolayer of the DCV‐iPr and DCV‐Me systems, respectively, placed on a graphite surface. A schematic representation of vacuum deposition simulations is shown for DCV‐iPr in panel (a). Panels (c) and (d) present side views of the thin‐film morphologies of the two systems at the end of the deposition simulation. The breakdown of different layers, as mentioned in the text, is illustrated by representing each layer in a different color. L1 includes molecules whose center of mass (COM) is within 3.5 Å from the substrate. Each subsequent layer (L2, L3) consists of molecules whose COM is within 3.5 Å of the previous layer. Panel (e): Correlation between the intermolecular distance (R) and the angle (θ) for molecules in the indicated layer and their closest neighbors in the upper layer for DCV‐iPr and DCV‐Me depositions. Panels (f) and (g) represent the correlation between *R* and θ between the first two layers (L1 and L2) and the second and third ones (L2 and L3), respectively.

Thin film structures were generated through an automated method for the simulation of vapor deposition [25, 26, 27]. Molecules were deposited on a planar crystalline monolayer obtained by replicating the experimental cell for DCV‐iPr and the SCSP predicted cell for DCV‐Me. The template monolayer was placed on a flat graphite surface (Figure 3a,b) and equilibrated at the deposition temperature. Such architecture allows our system to resemble a real device section far from the interface substrate/molecule, where the effect of a bottom rough surface (e.g., C_60_) is less evident, and the molecules begin to organize as a planar thin film. For each deposited layer, molecular diffusive processes are expected to favor a 2D crystalline configuration in accordance with the underlayer morphology, by means of π‐stacking interactions, and reflecting the organization of the first layer template. Indeed, we observe this tendency occurring in both systems, albeit at a different extent: for DCV‐iPr, the “induced morphology” effect begins to fade already at the third layer, while for DCV‐Me it propagates until the last deposited layer. This difference is clearly visible when looking at the overall morphology of the final deposition snapshots (Figure 3c,d): while the top section of the DCV‐iPr system shows a quasi‐amorphous molecular disposition, for DCV‐Me it still resembles the first layer organization. The larger hindrance of the i‐Pr moiety for DCV‐iPr, which hampers the molecular diffusion over the sample, explains this difference in the deposition outcome and the decrease in crystallinity with the distance from the first monolayer. However, it is also likely that these simulations tend to favor glassy organizations of the molecules because of the limited simulation times that prevent the crossing over high energy barriers (as, for instance, large amplitude motion in buried layers). In other words, we expect our vapor deposition protocol to overshoot the amount of positional disorder and underestimate spatial correlation lengths (estimated to be in the range 6–8 nm from GIWAXS).

A first assessment of the possible influence of intermolecular interactions on the optical properties of the films can be gained at this stage, noting that excitonic interactions are expected to switch from H‐like (>0) to J‐like (<0) as the relative longitudinal shift of the molecular transition dipoles (Figure S7) increases from a cofacial to a displaced arrangement. We thus recorded the following metric on the deposited samples: The distance R between each molecule and its closest neighbor in the upper layer was correlated to the angle θ between the distance vector and its projection on the xy plane (Figure 3e). Thus, large R combined with small θ values should favor J‐like couplings, while small R and large θ would lead to H‐like couplings. Figure 3f,g clearly show that the nearest neighbor interactions between the first two monolayers correspond to the first case (J‐like) in DCV‐iPr but to the second case (H‐like) in DCV‐Me. When moving away from the surface, the simulations suggest that this specific organization fades away in the case of DCV‐iPr in line with the increased positional disorder discussed above.

### 2.4 Simulation of Thin‐Film Optical Absorption and Emission

#### 2.4.1 H‐ Versus J‐aggregation

In the previous section, we have demonstrated that DCV‐iPr and DCV‐Me arrange differently, both in their bulk crystalline and thin‐film morphologies generated following the deposition process. As extensively discussed in the literature [17, 18, 19], the different arrangements of the molecules in the solid‐state impact the strength and sign of excitonic interactions, $V_{kl}(R_{\mathrm{lf}}(t))$, between singlet excitations localized on the molecules, the so‐called Frenkel excitons (FE). These interactions, in turn, can significantly influence the delocalization of excitonic eigenstates and, consequently, the optical response as the system transitions from solution to the solid state (see Methods).

To understand which is the predominant H‐ versus J‐like character of DCV‐iPr and DCV‐Me systems, we started by computing the excitonic interactions between nearest‐neighbor pairs of molecules k and l extracted from supercells of both systems generated by replicating the related unit‐cells in Table 1 (see details in Methods and Section S9). To compute these couplings, reported in Table S10 for DCV‐iPr and Table S11 for DCV‐Me, we employed the multi‐state diabatization method called fragment excitation energy difference–fragment charge difference (MS‐FED‐FCD) in conjunction with TDDFT [34, 35, 36]. As previously discussed, this approach provides an accurate reference for excitonic interactions [19, 37]. While the magnitude of J‐like interactions remains similar in both systems, we found that both systems exhibit sizable H‐like (side‐by‐side, positive) and J‐like (head‐to‐tail, negative) couplings, with the former being stronger in magnitude in both cases. Notably, the smaller steric hindrance of the methyl group in DCV‐Me allows the molecules to come closer together (see, for instance, D1 and D6 pairs in Figure S16), resulting in H‐like couplings more than twice as strong as those in DCV‐iPr. This already hints to a stronger H‐like effect on the optical spectrum of DCV‐Me compared to that of DCV‐iPr. To extend this observation to realistic nanometer‐sized samples, it is important to first recognize that excitonic interactions are inherently long‐range (a characteristic often necessary to consider for an accurate description of the states of extended systems [37, 38]). Second, excitonic couplings $V_{kl}(R_{\mathrm{lf}}(t))$ are modulated by the intermolecular arrangement of the molecules, which varies due to (generally low‐frequency) molecular motions, as reflected in their dependence on the nuclear coordinates $R_{\mathrm{lf}}(t)$ in real morphologies and along molecular dynamics trajectories. To account for all these factors and enable the calculation of several thousand coupling points, a more computationally efficient method than MS‐FED‐FCD is needed, as discussed below. Before concluding, we note that the MS‐FED‐FCD method also enables the calculation of couplings between localized Frenkel exciton states and intermolecular charge transfer (CT) states within the same molecular domains. These photo‐induced hole and electron charge transfer interactions decay exponentially with the distance between the involved molecular orbitals. These interactions are significant only for a few molecular pairs with particularly favorable stacking arrangements (Tables S12 and S13) and are neglected from now on.

**FIGURE 4:**
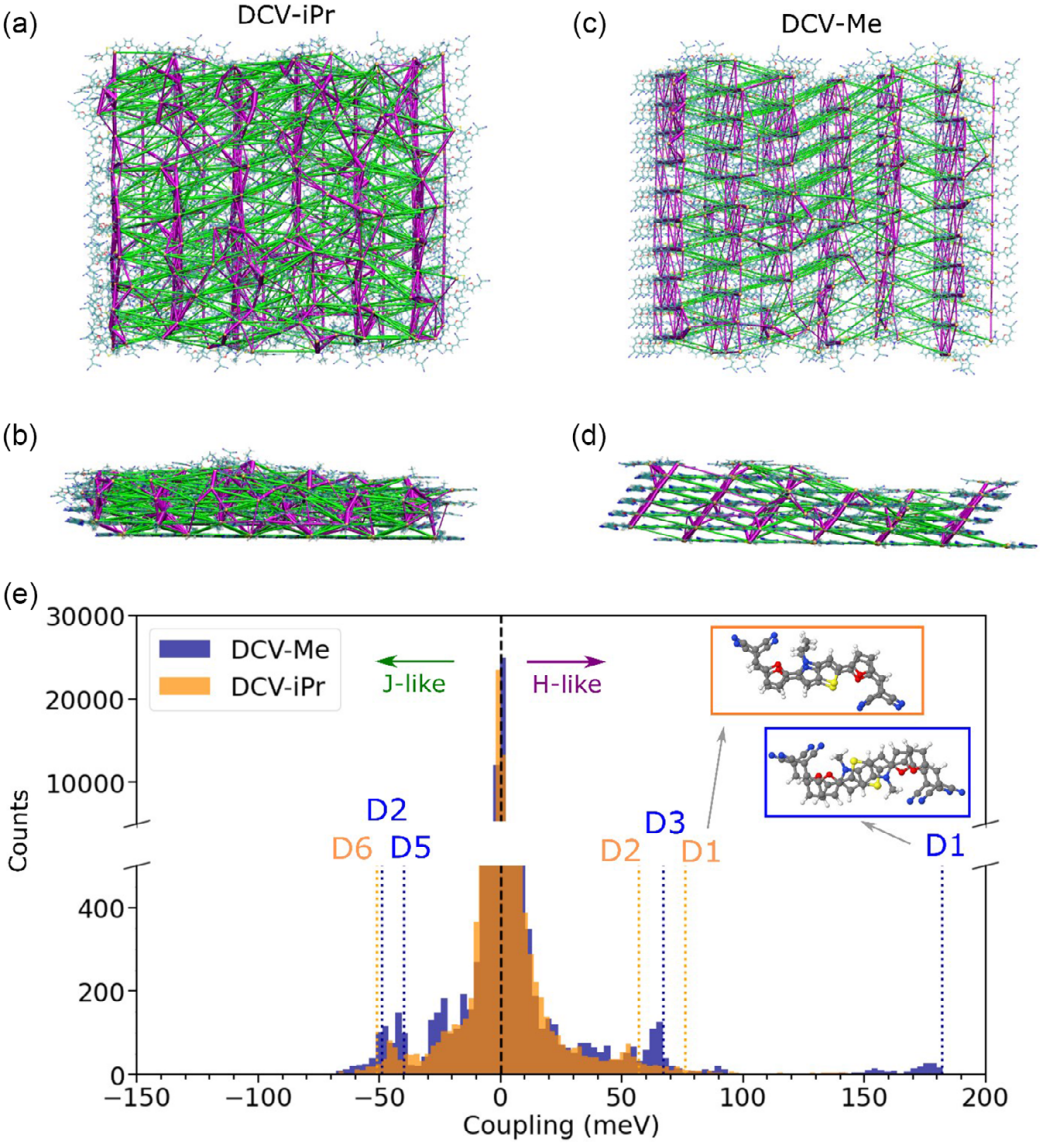
Excitonic couplings network for DCV‐iPr and DCV‐Me thin‐films represented from a top view in panels (a) and (c), respectively; and lateral view (b) and (d), respectively. H‐like (J‐like) interactions are shown as green (magenta) segments. Excitonic couplings are computed as detailed in Methods and Section S9. Note that the sign of the couplings, positive (negative) for H‐like (J‐like) interactions, is consistently given to all the molecular pairs. Only interactions stronger than 15 meV are shown for clarity purposes in the network maps, and the thickness of the colored lines is proportional to the excitonic coupling strength. (e) Coupling distributions for the two systems, with green (magenta) arrows pointing to H‐like (J‐like) interactions. Vertical dashed lines represent, for reference purposes, the excitonic coupling values of three of the most strongly interacting molecular pairs present in the perfect crystal (see Tables S10 and S11). Note in the inset that, in contrast to DCV‐Me, D1 is not a p‐stacking dimer for DCV‐iPr, but the two involved molecular planes have a vertical distance of >6.8 Å since there is another shifted molecule in between the two molecules forming D1. Accordingly, the D1 coupling (${\tilde{V}}_{kl}$) is much smaller for DCV‐iPr than for DCV‐Me.

The full set of excitonic interactions between FE states can be computed using transition electrostatic potential (TrESP) charges calculated as explained in Methods and Section S9. This approach allows us to compute excitonic interactions using a simple Coulomb sum (Equation S5) and to keep the sign of the interactions among all different pairs consistent. In Figure 4a–d, we visually present the excitonic coupling network characteristics of the deposited thin‐film structures of DCV‐iPr and DCV‐Me, while similar maps for the crystalline phase are provided for two representative supercells of both systems in Figure S18. These maps quantify the strength and sign of the excitonic interactions characterizing the structures of the two systems: both exhibit numerous J‐like and H‐like interactions, depicted in green and magenta, respectively.

To illustrate the influence of molecular order on excitonic interactions across different length scales, we will discuss the evolution of the simulated spectra with the number of monolayers for both the crystalline and the simulated vacuum‐deposited films. J‐like interactions dominate within the first monolayer for both DCV‐iPr and DCV‐Me, as quantified by the total H‐to‐J interaction ratio in Table 2. The layer definitions are provided in Figure 3c. The most significant difference between the two systems appears at the bilayer stage, which, according to the simulations, remains largely ordered in both vacuum‐deposited structures. At this point, DCV‐Me already exhibits a clear H‐type character, while DCV‐iPr is still dominated by J‐type interactions. As additional layers are included, the H‐like character becomes increasingly pronounced in both model systems, as also visible in Figure 4c–d.

**TABLE 2:** Percentages of H‐ versus J‐like interactions across the different layers of the simulated vacuum deposited structures.

System	Layers^a^	% H‐like^b^	% J‐like^b^	Ratio^c^
DCV‐Me	1	43.4	56.6	0.77
	1,2	52.6	47.4	1.11
	1 to 3	55.3	44.7	1.24
	1 to 4	56.5	43.5	1.30
	All	57.9	42.1	1.38
DCV‐iPr	1	47.1	52.9	0.89
	1,2	47.8	52.2	0.92
	1 to 3	54.4	45.6	1.19
	1 to 4	55.5	44.5	1.25
	All	56.3	43.7	1.29

a A graphical representation with related definition of the different layers is reported in Figure 3. Layer 1 includes only the first layer of molecules, considering molecules whose center of mass (COM) is within 3.5 Å of the substrate; layer 1,2 both the first and second layers and so on.

b The amount of H‐like vs J‐like interactions is calculated using the signed couplings (see Methods) as 
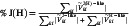
 .

c H‐to‐J interactions ratio computed as: 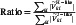. Only the final MD structure of each system has been considered for this statistical analysis.

The total coupling distribution computed for ∼50,000 interactions between all molecules in all the layers of a single representative MD snapshot of the vacuum‐deposited structures is shown in Figure 4e. As a first observation, it is clear that the vacuum‐deposited structure still maintains a good degree of order, as noticeable when comparing the peaks in the distribution and the values of the couplings in the crystal phases. Interestingly, as discussed earlier, the distribution reveals that the less hindered chemical structure of DCV‐Me results in a tightly packed, ordered arrangement in the thin film (Figure 4a,b), which helps maintain strong H‐like interactions between layers—reaching up to 180 meV—even in the deposited structure, closely matching the value observed in the crystal form (see dashed vertical lines). In contrast, the bulkier nature of DCV‐iPr reduces both the number and strength of H‐like interactions, instead promoting equally strong J‐like interactions, leading to a balance between the two. Additionally, we note that the majority of long‐range, weak (<5 meV) excitonic interactions are H‐like for DCV‐Me and J‐like for DCV‐iPr. The significant impact of these observations on the optical properties of both systems will be explored in the next section.

#### 2.4.2 Energetic Disorder

Another important ingredient that can significantly affect the delocalization of the excitonic states in molecular aggregates is the magnitude of the energetic disorder, i.e., the fluctuations of excitation energies $E_{S_{0}\to S_{1}}^{\text{film},k}(R(t))$ in the excitonic Hamiltonian, Equation (1). In fact, the energy of the localized excitation on the *k* molecule is influenced by the polarizable embedding of the other molecules within the thin‐film aggregate. Notably, the dependence on the nuclear coordinates, R(t), indicates that the excitation energies also depend on the conformational arrangement, as well as vibrational dynamics of the given molecule over time. The total energetic disorder is, therefore, quantified by the standard deviation, σ, of the energy as a function of nuclear coordinates and time, and can be broken down into two main components [39, 40]: conformational ($\sigma_{\text{conf}}$) and environmental ($\sigma_{\mathrm{env}}$). $\sigma_{\text{conf}}$ depends on the intramolecular dynamics of molecules (affecting their excitation energies) and was estimated by computing the TDDFT excitation energies of representative molecules extracted from the thin‐film along the MD trajectory by evaluating the autocorrelation function of the vertical energy gap (details in the Methods). As shown in Table 3, we find that the conformational disorder is similar for both DCV‐iPr and DCV‐Me molecules. This is somehow expected given the similar structures and dynamics of the two molecules. Furthermore, the fluctuations of individual molecular excitation energies are mostly static over the timescales explored. We do, however, find a weak dependence of these excitation energies with respect to the position along the vertical stacks, with (as expected) slightly lower excitation energies and narrower distributions in the inner versus outer layers. This difference is associated with positional disorder, which is greater in the outer layers than in the inner layers.

**TABLE 3:** Excitation energies and disorder (all elements in eV).

System	Layers^a^	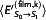 b	⟨Δ 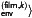	$\sigma_{\mathrm{env}}$ c	$\sigma_{\text{conf}}$ d
DCV‐Me	1	2.367	−0.066	0.019	0.045
	1,2	2.375	−0.063	0.019	0.047
	1 to 3	2.379	−0.067	0.022	0.056
	All	2.385	−0.065	0.024	0.058
DCV‐iPr	1	2.359	−0.067	0.015	0.041
	1,2	2.359	−0.068	0.017	0.043
	1 to 3	2.366	−0.062	0.022	0.048
	All	2.385	−0.050	0.028	0.049

a The representation of the systems with related definition of the different layers is reported in Figure 3.

b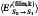 is calculated as the ensemble average difference between the excitation energy of the molecule in vacuo plus the negative environmental energy computed for each molecule: 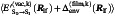.

c The $\sigma_{\mathrm{env}}$ is estimated as: $\sqrt{\left\langle ({\text{Δ}}_{\left(\text{film},k\right)}^{\mathrm{env}}(R_{\mathrm{lf}})-\langle {\text{Δ}}_{\left(\text{film},k\right)}^{\mathrm{env}}{\left(R_{\mathrm{lf}})\rangle \right)}^{2}\right\rangle }$. Both the ensemble average and the standard deviations are taken considering the molecules of the last equilibrated MD snapshots of the thin‐film structures of both systems.

d The $\sigma_{\text{conf}}$ is calculated as 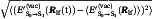 where the ensemble average is performed over the energies of all the molecules extracted from the last MD snapshots and those computed along time (after filtering the high frequency modes as explained in Methods). $R_{\mathrm{lf}}$ represents the degrees of freedom associated with low‐frequency motions. We note also that the structures of the last MD snapshots considered were suitably minimized at the MM level, as described in Methods, to prevent high‐frequency modes, already treated at the quantum level, from contributing to the diagonal energetic disorder.

Each molecule is also subject to environmental electrostatic effects (${\text{Δ}}_{\mathrm{env}}^{\left(\text{film},k\right)}$), namely polarization and induction effects induced by the presence of the neighboring molecules [39, 41, 42]. Such environmental responses cause the vertical excitation energy in the solid state to decrease with respect to the value in vacuo. The standard deviation $\sigma_{\mathrm{env}}$ of the electrostatic energy is essentially static in time, as demonstrated for other organic systems in Ref. [39, 40]. This is because molecules within the thin film, once the deposition process has finished, move only slightly around their equilibrium positions without reorienting their electrostatic dipoles. This motion only minimally perturbs the environmental response [39, 40]. To obtain the response on each of the molecules, embedded polarizable microelectrostatic (ME) calculations for the *k* molecules taken from the last MD snapshots were performed as described in Ref. [40] and detailed in Methods. Interestingly, as for its conformational counterpart, the environmental disorder $\sigma_{\mathrm{env}}$ evolves as more layers are included, as a result of the increasing positional disorder away from the perfect crystal‐like organization in layers close to the film‐air interface compared to the inner layers in contact with the substrate. Finally, we note that diagonalizing the electronic Hamiltonian—including both diagonal and off‐diagonal disorder induced by low‐frequency fluctuations—yields a total disorder of 0.112 eV for DCV‐Me and 0.089 eV for DCV‐iPr. This indicates that a broader spectral linewidth can be expected for DCV‐Me compared to DCV‐iPr.

#### 2.4.3 Aggregate Steady‐State Spectra

We now investigate in detail the key spectral changes characterizing DCV‐iPr and DCV‐Me upon aggregation. Using the computed excitonic couplings and excitation energies, we constructed the electronic Hamiltonian reported in Equation (1) for both DCV‐iPr and DCV‐Me vacuum‐deposited thin‐film phases (see structures in Figure 3). All sources of disorder entering the Hamiltonian affect the spatial localization of the states, as well as the coupling between the electronic and nuclear degrees of freedom. This exciton‐phonon coupling is necessary to have a faithful reproduction of the spectral vibronic shape of the steady state spectra of both DCV‐derivatives. Notably, while we have previously used such a Hamiltonian and a similar protocol to describe the optical properties of crystalline samples [19, 29], here we apply it, for the first time, to samples generated from atomistic vapor deposition simulations. Emission PL spectra are modeled assuming full thermalization in the excitonic density of states, thus with contributions to the spectrum from the lowest aggregate excited states weighted by their thermal occupation at room temperature.

**FIGURE 5:**
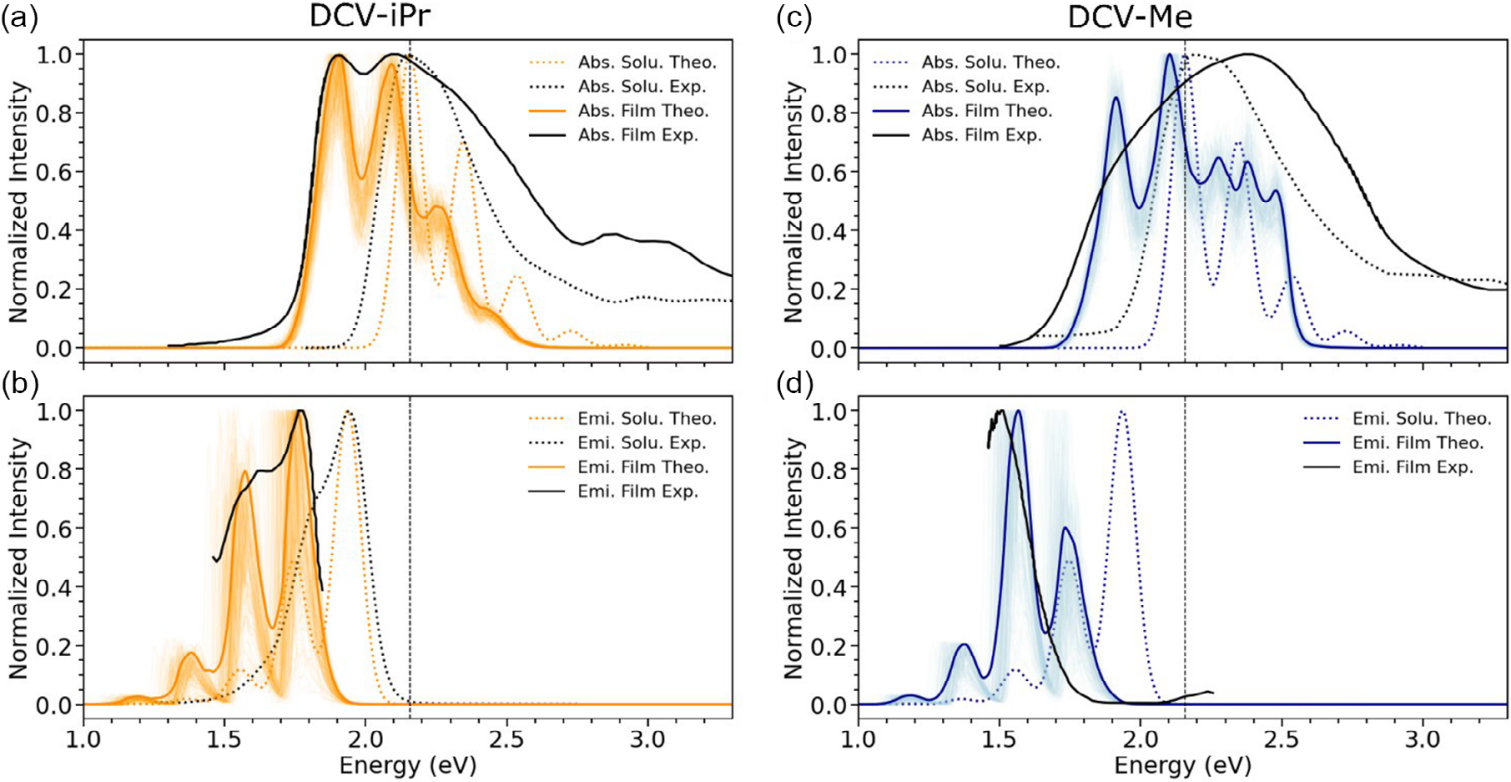
Comparison of computed vs experimental absorption spectra in panel (a) and (c), and emission spectra in (b) and (d) for DCV‐iPr and DCV‐Me, respectively. Experimental absorption and emission in solution are represented with dashed black lines, while the corresponding thin‐films data are depicted with solid black lines. Computed absorption and emission spectra related to the thin‐film morphologies of DCV‐iPr and DCV‐Me, represented with solid orange and blue lines, respectively, are obtained as an average spectrum over 100 realizations of diagonal and off‐diagonal disorder as explained in the text. The computed absorption and emission spectra for both systems are uniformly shifted by −0.38 eV to correct the remaining DFT inaccuracy. Individual contributions of the different realizations are shown with shaded lines by applying a small convolution of 10 meV to the sticks. Computed absorption and emission spectra related to the single isolated molecule are calculated as discussed in Section 2.2 and reported here for reference. Vertical dashed lines represent the absorption maximum in the solvent for the DCV‐derivates as a reference.

The Hamiltonian is constructed and diagonalized for each realization of the disorder (both on energies and couplings), and the resulting excitonic states are used to generate the optical absorption and emission spectra via Equations S3, S12, respectively. The ensemble average spectrum is presented with thick solid lines in Figures 5a,c for DCV‐iPr and DCV‐Me, respectively, while thin transparent lines are the spectra of individual simulation frames. In both systems, the theoretical simulations capture reasonably well the main changes in spectral envelope between thin‐film DCV‐iPr and DCV‐Me. In the case of DCV‐iPr, the balanced contributions from H‐ and J‐like interactions yield: (i) intensities that are very similar for the purely electronic 0–0 and vibronic sideband 0–1 transitions; and (ii) excitonic density of states with optically allowed transitions on the low‐energy side, translating into spectrally resolved spectral features and a small Stokes shift. Both theoretical findings are in good agreement with experiment and reasonably robust against small variants in the simulation protocol. For instance, the same conclusion holds if, instead of the positionally disordered thin films prepared by vapor deposition simulations, we consider translationally equivalent molecules as in the perfect crystal, Figure S18. Here, the bands are slightly narrower and more defined due to the smaller amount of disorder.

Notably, the calculations also nicely reproduce the (small) measured Stokes shift and, therefore, also the (low) energetic disorder in the films (see Figure 5a,b). The situation changes drastically in DCV‐Me (Figure 5c,d). There, the balance in the excitonic interactions is largely biased towards positive, H‐like couplings. As a result, there is a massive shift of the optically allowed excitonic transitions towards high energy and a large spectral Stokes shift between absorption and PL maxima, both effects captured by the model.

In Figures S19 and S20, we observe a clear evolution of the spectral features with the number of layers deposited on the substrate for both DCV‐iPr and DCV‐Me. As shown in Table 2, increasing the number of layers enhances the prevalence of H‐like interactions, which translates into a growing intensity of the 0–1 band in both absorption and emission, along with an increasing Stokes shift. This trend characterizes both crystalline and vacuum‐deposited morphologies and it is consistent with what we observed experimentally in Figure S21 when increasing the deposition rate. The comparison between the crystalline and vacuum‐deposited morphologies reveals that short‐range crystalline order is beneficial for maintaining a dominant 0–0 transition (as seen in the comparison at the four‐layer level), while long‐range order becomes detrimental, as indicated by the increasing weight of the 0–1 transition in more ordered systems with 8–10 layers.

We conclude that the spectral optical lineshapes of the two films provide a direct fingerprint for distinct molecular organization in the two materials: even if the conformational disorder and the electrostatic environment is small and similar for the two systems, mixed HJ interactions offering a narrow, low‐energy, distribution of light‐emissive states in DCV‐iPr against blue‐shifted excitonic absorption and a broad distribution of low‐energy darker states in DCV‐Me.

### 2.5 Analysis of the Voltage Losses

For good solar cells, steep absorption onsets are desired, minimizing the energy loss for strongly absorbed photons, and the narrowest DCV‐iPr absorption spectrum with respect to DCV‐Me explains the better performances of the corresponding solar cells (Table 4). Indeed, the difference between *q*V_oc_ (0.91 and 0.88 eV, respectively) and the energy at which the absorption peaks (*E*_p_, 1.89 eV and 2.40 eV) is significantly smaller for DCV‐iPr:C_60_ as it is for DCV‐Me:C_60_. Still, the DCV‐iPr:C_60_ system is not particularly efficient and we therefore carried out a detailed investigation of its voltage losses: We measured the sub‐gap part of the photovoltaic external quantum efficiency (EQE_PV_) spectrum by Fourier‐transform photocurrent spectroscopy (FTPS) [43], to reveal the most important factors determining the energy loss for strongly absorbed photons, i.e., the difference between the photon energy where absorption peaks and *q*V_oc_.

**TABLE 4:** Photovoltaic performances of solar cells of both investigated materials.

Active layer	PCE (%)	J_sc_ (mA/cm^2^)	FF	V_oc_ (V)
DCV‐iPr:C60 (30 nm)	8.2 (7.6 after MMC)	13.0 (12.0 after MMC)	0.69	0.91
DCV‐Me:C60 (30 nm)	4.6	10.1	0.52	0.88

**FIGURE 6:**
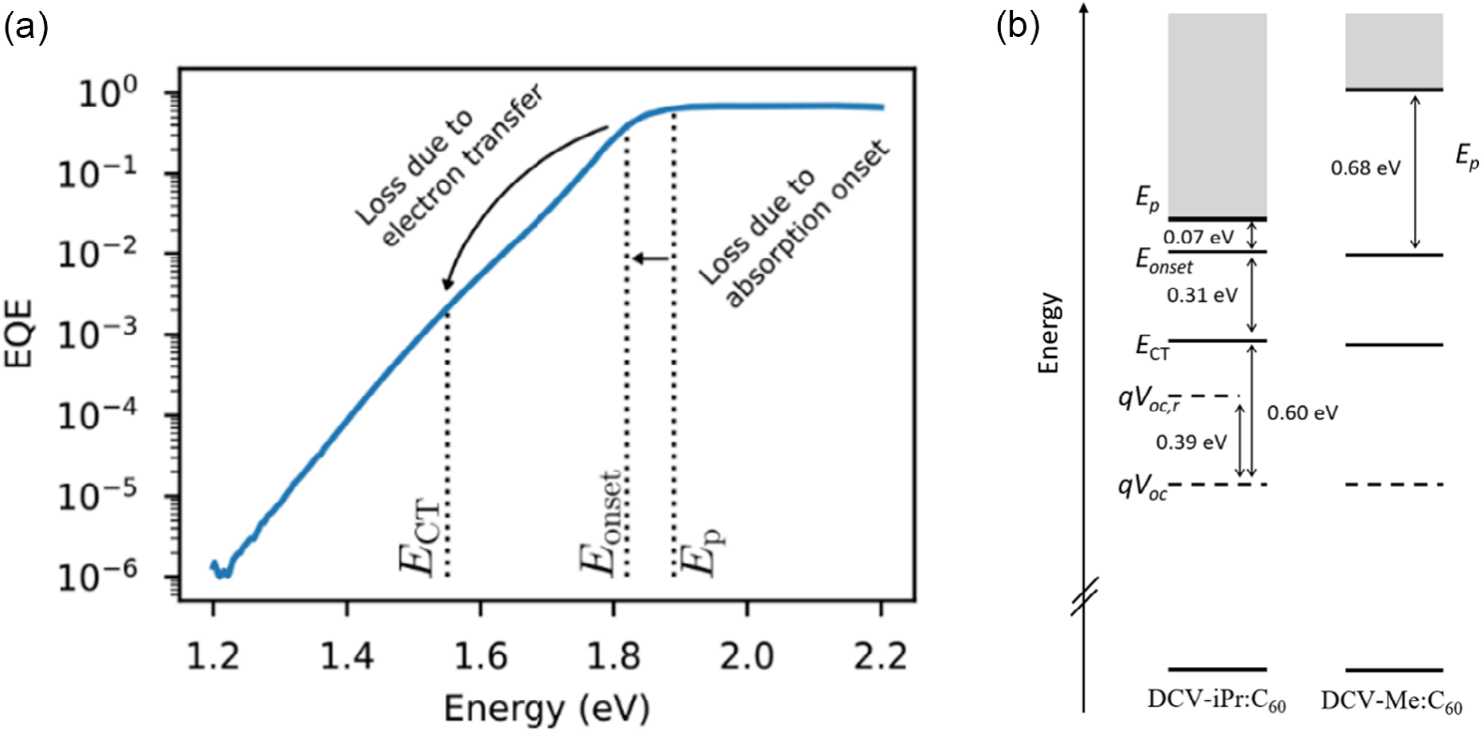
(a) Sensitively measured EQE spectrum by Fourier‐transform photocurrent spectroscopy (FTPS) of the DCV‐iPr:C_60_ device, plotted on a logarithmic scale. The neat donor absorption onset energy *E*_onset_, peak energy *E*_p_, and CT state energy (*E*_CT_) are indicated. (b) Breakdown of photon energy losses and voltage losses for strongly absorbed photons in DCV‐iPr:C_60_ and DCV‐Me:C_60_ blends. The region of strong absorption is indicated in gray.

The subgap EQE spectrum (Figure 6a) shows, below the absorption peak *E*_p_ at 1.89 eV and the absorption onset *E*_onset_ at 1.82 eV, a broad low intensity absorption band, which we ascribe to charge transfer (CT) absorption. The energy of the CT state (*E*_CT_) is determined via the fitting method described in Ref. [21] to be 1.51 eV. The onset energy *E*_onset_ was determined as the intercept between the reduced PL and absorption spectrum of the neat donor material [44]. Figure 6b summarizes the photon energy and voltage losses experienced by strongly absorbed photons. As described above, the *E*_p_−*E*_onset_ difference for our systems is entirely determined by molecular packing. From the optical spectra in Figure 1, we deduce an *E*_p_−*E*_onset_ of 0.07 and 0.68 eV for DCV‐iPr and DCV‐Me, respectively, in going from balanced HJ‐like aggregation to more H‐like aggregation. Further energy loss is due to charge transfer at the D–A interface, quantified by taking *E*_onset_−*E*_CT_, which amounts to 0.31 eV in the case of DCV‐iPr:C_60_. The remaining *E*_CT_−*q*V_oc_ loss is due to radiative and non‐radiative recombination. The non‐radiative part of this loss is quantified using the method described in Ref. [45] and amounts to 0.39 eV.

Then, as is the case for the best solution‐processed organic solar cells, also for the DCV‐iPr:C_60_ state‐of‐the‐art vacuum‐deposited organic solar cell, the main source of the total voltage loss for a strongly absorbed photon is thus the non‐radiative recombination loss, constituting a bit less than half of the total loss *E*_p_−*q*V_oc_ of 0.98 eV. A large part of these losses is due to charge transfer at the donor/acceptor interface (*E*_onset_−*E*_CT_) constituting around 1/3 of the total loss. In contrast, in the case of DCV‐Me:C_60_, the largest contribution to the total loss of around 1.6 eV originates from the shallow absorption tail. In both cases, minimizing losses due to charge transfer at the donor/acceptor interface (*E*_onset_−*E*_CT_) would increase the V_oc_ by 0.31 V, up to 1.12 V for DCV‐iPr:C_60_. Keeping all other photovoltaic parameters (J_sc_ and FF) the same, this would result in a power conversion efficiency of 9.3%. Reducing non‐radiative voltage losses from 0.39 V to typical values achieved today for solution‐processed OPV of about 0.25 V would further increase efficiency beyond 10%. Further increased efficiencies will require higher charge carrier mobilities and thus increased FF values. The major difference between the two active layers is thus the *E*_p_−*E*_onset_ difference. This affects photovoltaic device performance as follows: the largest *E*_p_−*E*_onset_ in the DCV‐Me:C_60_ device results in less overall solar photon harvesting and thus a reduction in maximum achievable J_sc_, even though the V_oc_ remains almost unchanged.

An estimation for the apparent Urbach energy and the Urbach energy of the CT shoulder at room temperature can be calculated for DCV‐iPr:C_60_ from Figure 6a. The following values are extracted: $E_{U,app}=47$ meV and $E_{U,CT}=42$ meV (Figure S24). While the apparent Urbach energy is comparable to reported Urbach energies of commercial a‐Si:H thin film solar cells ($E_{U}\approx 47$ meV) and solution‐processed PM6:Y25 devices ($E_{U}=48.5$ meV) [22, 23], the Urbach energy of the CT shoulder is significantly smaller. On the other hand, a tail energy above *k*_B_T is not yet ideal for efficient solar cells and indicates harmful influence of both intermolecular and intramolecular disorder, which negatively affects both V_oc_ and carrier mobility and thus FF.

### 2.6 Efficient OPV Devices With BHJ + Architecture

**FIGURE 7:**
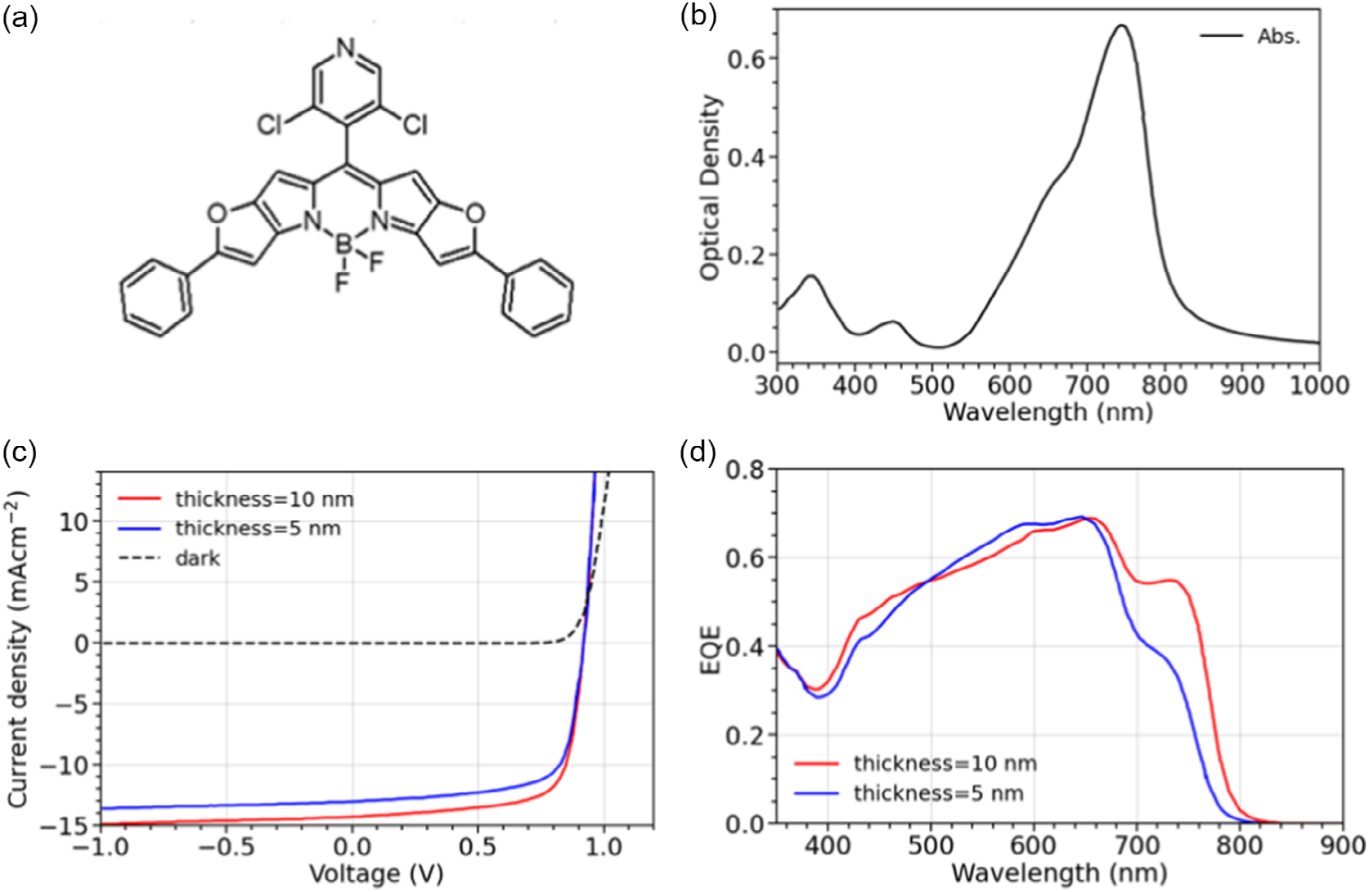
(a,b) Chemical structure and absorption of 30 nm film of BODIPY‐Pyr‐Cl_2_ on quartz glass substrate. (c) J–V‐characteristics measured with mask for 5 and 10 nm BODIPY‐Pyr‐Cl_2_ active layer thickness in a BHJ + device with DCV‐iPr:C_60_. (d) Measured EQE spectra of BHJ + devices with DCV‐iPr:C_60_ (30 nm)/ BODIPY‐Pyr‐Cl_2_ (5/10 nm) as active layers.

An efficiency of 8% for a single heterojunction of DCV‐iPr:C_60_ is an acceptable value but still below the state of the art for vacuum deposited OPV. To improve the performance, the so called “BHJ +” architecture was applied. The layer structure of the BHJ + device is ITO / C_60_ (15 nm) / DCV‐iPr:C_60_ (30 nm) / BODIPY‐Pyr‐Cl_2_ (5/10 nm) / TaTm (10 nm) / TaTm:NDP‐9 (30 nm) / NDP‐9 (1 nm) / Au (5 nm) / Al (100 nm). The DCV‐iPr:C_60_ layer was deposited at optimal mixing ratio and substrate temperature. The BODIPY‐Pyr‐Cl_2_ layer was deposited on top of the mixed DCV‐iPr:C_60_ layer. In general, BHJ + is a n‐i‐p device configuration with an intrinsic absorber layer comprising first a layer of a bulk heterojunction (BHJ) and second a thin pristine donor‐type absorber layer forming a planar heterojunction (PHJ) with the acceptor component of the BHJ layer. The advantage of such architecture is that efficiency can be increased by enhancing the generated photocurrent by absorption and exciton diffusion in the pristine layer without losing FF and V_oc_. For this purpose, the PHJ‐material must fulfill several requirements: high exciton diffusion length as well as sufficiently high hole mobility to enable efficient exciton dissociation and charge transport, similar donor‐HOMO level to that of the BHJ layer material so as not to lose V_oc_, and preferably complementary absorption spectrum to harvest photons from a broad spectral range. Heliatek's proprietary NIR absorber material BODIPY‐Pyr‐Cl_2_ was used as a PHJ or plus‐layer in a BHJ + device in combination with DCV‐iPr:C_60_ BHJ. BODIPY‐Pyr‐Cl_2_ is a BODIPY class material which is somewhat similar to the materials reported by Li et al. in Ref. [46]. The BODIPY core is extended at both sides with a furane ring to form a fused conjugated 5‐ring system. Finally, phenyl rings are laterally attached at the para‐position of the furane, which further extends the conjugated system since phenyl can arrange coplanar to furane [46]. However, in contrast to the materials mentioned by Li et al., BODIPY‐Pyr‐Cl_2_ bears o, o‐dichloropyridine in the meso position. Due to the steric demand of the two chloride atoms, the dichloropyridine is expected to be largely orthogonal to the BODIPY core. The chemical structure and the corresponding absorption spectrum of a 30 nm BODIPY‐Pyr‐Cl_2_ thin film are presented in Figure 7a,b, respectively. BODIPY‐Pyr‐Cl_2_ has a strong absorption in the NIR spectral range with a maximum at 743 nm and sharp absorption edge (steepness of 10.3 eV^−1^). In PHJ architecture, BODIPY‐Pyr‐Cl_2_ devices (see Table S15 and Figure S22) deliver V_oc_ of 0.97–0.98 V. Fill factors of around 77% for 8–12 nm active layer and a continuous photocurrent increase with layer thickness up to at least 12 nm (Figure S15) indicate moderately high exciton diffusion length and good hole mobility of BODIPY‐Pyr‐Cl_2_.

Figure 7c shows the J–V‐characteristics measured with mask of BHJ + devices comprising 5 and 10 nm BODIPY‐Pyr‐Cl_2_ plus‐layers in combination with a 30 nm BHJ layer of DCV‐iPr:C_60_. The devices deliver open circuit voltage of 0.92 V and FFs of 71% and 72%, respectively. The current density increases from 13 to 14.2 mA/cm^2^ with increasing plus‐layer thickness, as can be seen from the EQE spectra in Figure 7d. Additionally, comparison to Figure S1 shows that the enhanced J_sc_ of the BHJ + device is due to extended absorption range above 700 nm and thus originates from excitons generated in the BODIPY‐Pyr‐Cl_2_ layer. The fact that there is no FF loss upon increasing BODIPY‐Pyr‐Cl_2_ thickness confirms good exciton diffusion length and transport properties of the NIR absorber. The efficiency of the BHJ + devices after spectral mismatch correction is 8.5% for 5 nm BODIPY‐Pyr‐Cl_2_ plus‐layer and 9.5% for 10 nm plus‐layer, respectively. The presented results show the potential for efficiency optimization of vacuum‐deposited small‐molecules organic solar cells with C_60_ as the acceptor.

## 3 Concluding Remarks

Vacuum deposition enables, in principle, precise control over film thickness, morphology, and composition, potentially leading to high‐quality, defect‐free layers. However, designing vacuum‐processed organic solar cells remains challenging, as it requires balancing the energetic and morphological requirements for efficient photovoltaic performance with the need for organic semiconductors that remain stable under vacuum conditions. The ideal molecular donors and acceptors should minimize voltage losses by exhibiting steep absorption both at the molecular scale (through molecular rigidity and conformational stiffness) and in evaporated thin films (via supramolecular organization).

Among the molecular donors synthesized at Heliatek GmbH, two structurally similar molecules, DCV‐Me and DCV‐iPr, exhibit strikingly different photovoltaic responses. To understand this contrast, we conducted a comprehensive experimental‐theoretical study combining crystal structure predictions, vapor deposition simulations, advanced excited‐state electronic structure calculations, and model Hamiltonian solutions with single‐crystal determination, GIWAXS investigations, photovoltaic response analysis, and voltage loss studies.

Our findings reveal that substituting a methyl group with an isopropyl group on the donor backbone introduces steric effects that shift molecules along their long molecular axes and increase intermolecular distances, weakening π–π interactions. This structural modification leads to a brick‐wall molecular arrangement, resolved in single crystals and preserved in thin films, which favors J‐like excitonic interactions. As a result, low‐lying bright electronic states emerge at the bottom of the excitonic density of states, producing spectrally resolved optical absorption and emission spectra with a reduced Stokes shift.

This work establishes design rules linking molecular packing to photovoltaic losses associated with donor absorption onset steepness and demonstrates how minor chemical modifications can significantly impact this parameter. While DCV‐iPr‐based solar cells outperform their DCV‐Me counterparts due to sharper optical absorption, further optimization is needed. The primary limitation in DCV‐iPr:C_60_ vacuum‐deposited organic solar cells arises from voltage losses caused by low‐energy charge–transfer excitations below the donor absorption onset. Addressing this issue requires replacing C_60_ with an acceptor featuring shallower electron absorption, reducing the energy offset for photoinduced charge transfer—an approach successfully applied in solution‐processed non‐fullerene‐acceptor polymer solar cells. Consequently, a promising direction for further efficiency improvement is the development of efficient, light‐absorbing, and evaporable acceptor molecules.

Other important questions deserve attention. The weakening of π–π interactions in the donor domains, while beneficial for light absorption and reducing voltage losses, may negatively affect transport properties, particularly the mobility of hole carriers. To address this, we propose the use of original BHJ + device architecture. Although this strategy has proven successful in the present work, further optimization will certainly be required. Ongoing efforts aim to assess potential charge‐transport bottlenecks in materials with sharp optical absorption. Finally, correlating alkyl chain structure (which directly affects thermal transition temperature (*T*_g_), molecular packing, and diffusion behavior) with operational stability in vacuum‐processed OSCs is highly meaningful for further improvements. Shorter or less flexible side chains that increase *T*_g_ and reduce molecular mobility are expected to suppress diffusion‐driven morphological evolution, thereby improving long‐term device stability [47, 48, 49]. Therefore, further investigation along these lines is strongly encouraged.

## 4 Methods

### 4.1 Device Fabrication and Characterization

All organic thin films, as well as all organic solar cells, were deposited at ultrahigh vacuum conditions in a vacuum system with 10^−7^ mbar base pressure made by Creaphys GmbH, Dresden, Germany. All organic semiconductors were thermally evaporated from ceramic boats at rates of 0.2 Å/s. Rates and film thickness were measured by calibrated quartz microbalances. Quartz glass substrates were used for all samples with optical measurements, while structured ITO‐coated glass substrates were used for solar cell devices. The substrates were cleaned subsequently with Resin Clean, ethanol, and de‐ionized water, with a final step of Opticlean polymer cleaning, which was removed immediately before insertion into the vacuum system.

The solar cell stacks contained: the acceptor material Fullerene C_60_ (acquired from Frontier Carbon Corporation); the home‐made absorber materials DCV‐iPr or DCV‐Me, and BODIPY‐Pyr‐Cl_2_ (with structures shown in Figure 1a,d and Figure 7 [50]); the hole transport material *N*,*N*,*N*′,*N*′‐Tetra([1,1′‐biphenyl]‐4‐yl)[1,1′:4′, 1″‐terphenyl]−4,4″‐diamine (TaTm) [51]; and p‐dopant NDP‐9. The latter two are proprietary materials of Novaled GmbH, Dresden, Germany. All organic semiconductors were used in gradient sublimed quality to guarantee highest purity standards. Single crystals of the molecule DCV‐iPr were grown in a gradient sublimation vacuum system from MBraun, Dresden, Germany. The sublimation was carried out in a vacuum of 2*10^−7^ mbar, and the temperature was 280°C. Crystallographic data of these single crystals were determined and described elsewhere [52]. A reference to the .cif file of DCV‐iPr can be found at the end of the document. Current‐voltage characterization of the DCV‐iPr and DCV‐Me solar cells was done under inert nitrogen atmosphere using a Steuernagel sun simulator which was calibrated with a Fraunhofer ISE reference silicon solar cell. The current–voltage (J–V) characteristics of the encapsulated BHJ + devices were recorded in an ambient atmosphere with a source measure unit 2602B‐L Sourcemeter (Keithley) under a sun simulator based on LED matrix (WaveLabs) with a deviation from AM1.5G of 5%, using an aperture mask of 2.28 mm^2^. For the EQE measurement, a calibrated setup based on xenon lamp, a monochromator (Bentham TMc300), and a lock‐in amplifier (MFLI Zürich Instruments) was used.

### 4.2 Optical Steady‐State Experimental Measurements

Optical measurements of DCV‐Me and DCV‐iPr in solutions were carried out in 10 × 10 mm cuvettes with dimethylacetamide as a solvent; absorption spectra were recorded on a Perkin Elmer Lambda XLS spectrometer. Absorption and photoluminescence measurements in the solid state were carried out on 30 nm thick DCV‐iPr or DCV‐Me films on quartz substrates. The absorption of the films in the UV–Vis spectral range was measured in transmission mode using a two‐beam spectrometer UV‐1800 (Shimadzu Corporation). Steady‐state photoluminescence (PL) emission spectra were recorded using Spectrofluorophotometer RF‐5301 (Shimadzu Corporation).

### 4.3 GIWAXS Measurements

Out‐of‐plane (1D) GIWAXS was performed at XRD 3003 (θ/θ‐device, GE Sensing & Inspection Technologies, Ahrensburg, Germany) with monochromatic Cu−Kα_1_ radiation (λ = 1.5418 Å), parallel beam geometry, and scintillation detector. Angle of incidence was set to 0.2°. Slit blends of 0.2 mm and 0.3 mm before sample and detector were used, respectively. For the estimation of the lowest possible out‐of‐plane scattering coherence length, the Scherrer equation was used without corrections for instrumental and geometric broadening effects [53]. 2D GIWAXS was performed using the Ganesha 300 XL+ (SAXSLAB, Copenhagen, Denmark) with monochromatic Cu−K_α_ radiation (λ = 1.54 Å), and a Pilatus 300k area detector (pixel resolution: 0.172 × 0.172 μm^2^). The angle of incidence was fixed at 0.2°, and the sample‐to‐detector distance was set to 110 mm. Alignment of the sample was performed at GISAXS conditions at a detector‐sample distance of 1060 mm. GIWAXS data analysis was done with a self‐developed python script to transform the detector data to a spherical projection (*q*_xy_, *q*_z_) [54].

### 4.4 Crystal Structure Predictions

#### 4.4.1 Bulk Modeling

To predict bulk crystal structures, a CSP procedure in three steps was used to funnel the search towards the expected structure, the first two steps using force field calculations, the last one DFT with dispersion (see details below): (i) A conformational search was performed on isolated molecules, systematically varying the five (DCV‐iPr) or four (DCV‐Me) torsion angles of the molecules and optimizing the geometry by molecular mechanics (MM) to generate all possible minima, which were sorted out based on energy (see Section S2). Despite the absence of solid‐state packing that can modify the energy ranking, this step allowed us to provide a series of conformational candidates of lowest energy to use in the subsequent steps. (ii) Each conformation retained was used to build polymorphs by a Monte Carlo simulated annealing (SA), where the crystal parameters and orientations of the molecules were allowed to change. Thousands of polymorphs in the five most common space groups among the 230 existing ones, i.e., C2/c, P‐1, P21/c, P212121, and P21, were generated and optimized by MM, redundant polymorphs were removed, and the remaining ones were sorted out in energy (see Section S7). (iii) For each space group investigated, the most stable polymorphs were retained, their structure was further refined at the DFT‐D level, and they were again sorted out in energy. The most stable polymorphs were selected for further analysis, notably X‐ray diffraction (XRD) simulations (see XRD section).

All calculations for bulk‐crystal structure predictions were done with Materials Studio [55]. Steps (i) and (ii) were performed using the Dreiding force field [56], following a careful reparameterization process [57]. Specifically, (i) the four torsional angles of DCV‐Me and the five torsional angles of DCV‐iPr (as illustrated in Figure S2) were reparametrized against MP2/6‐31G** calculations, (ii) all the exocyclic angles were adjusted using the values from the MP2/6‐31G**‐optimized lowest energy conformer in the gas phase and (iii) the van der Waals radius of the hydrogen atom was modified to 2.83 Å [58]. The atomic charges are ESP charges calculated on conformations optimized by MP2/6‐31G**. The MM geometry optimizations were performed with the Smart method with an RMS force criterion of 0.001 kcal mol^−1^Å^−1^. For isolated molecules, the long‐range non‐bonded interactions were turned off using the Spline algorithm, with spline‐on and spline‐off parameters set to 13 and 14 Å, respectively. For polymorphs, periodic boundary conditions were used, and long‐range non‐bonded interactions were treated by the Ewald method, whose parameters were optimized to reach an energy accuracy of $10^{-5}$ kcal mol^−1^. The polymorphs were generated by a Monte Carlo SA having one temperature cycle of ultrafine quality, which implies that the temperature was allowed to increase from 300 K up to maximum 150,000 K in order to overcome the high potential barriers inherent to the solid state. A heating factor of 0.025 and a cooling factor of 0.0005 were applied.

The polymorph structures were compared using their radial distribution functions (RDF) calculated up to 7.0 Å with 140 bins. A crystal similarity measure (CSM) was calculated from the difference between the RDF of two structures, giving a number between 0 and 1. All structures with a CMS below a defined tolerance, here 0.11, are assumed similar and the least stable polymorph is removed from the list. The DFT‐D geometry optimizations were made using the PBE correlation exchange functional and a correction of dispersive effects by the Tkatchenko‐Scheffler approach [59], with a plane wave basis whose cut‐off energy is 680 eV. The energy difference required for convergence was $2.010^{-5}$ eV/atom and the maxima of force, pressure, and displacement were 0.05 eV/Å, 0.1 GPa, and 0.002 Å, respectively.

#### 4.4.2 On‐Surface Modeling

The MYTHOS (Morphological surveY for THin‐films of Organic Semiconductors) python code [52], interfaced with NAMD [60] software for molecular dynamics calculations and VMD software for molecular visualization, was used to investigate the crystallinity of the two molecules in the proximity of the substrate. The program is available in GitLab [61]. All details about the method and workflow are attentively described in the specific manual.

To predict on‐surface crystal structures, High Oriented Pyrolytic Graphite (HOPG) was used as a surface. The General Amber Force Field (GAFF) was used to describe the HOPG intermolecular Lennard–Jones interactions, while electrostatic ones were neglected by assigning a neutral charge to each graphite carbon atom. The FF parameters and charges of the two molecules were the same as those used for bulk modeling. For both structures, four molecular torsion angles (Figure S2, S5) were restrained in order to ensure the exclusive presence of the conformer found in the experimental crystalline structure of DCV‐iPr along the on‐surface dynamics. To do so, a harmonic potential was applied for each of the four dihedrals, centered at the equilibrium angles (either 0° or 180°), to obtain the desired conformation. Force constant values ranged from 1 to 5  kcal/mol, depending on the torsion. During MD simulations, periodic boundary conditions (PBCs) were applied across the two directions (x, y) parallel to the surface. The Particle Mesh Ewald (PME) [62] method was used to compute the electrostatic forces across the PBC system, using a cutoff of 12 Å for the calculation in the direct space, as well as for truncating Lennard–Jones interactions. The temperature was kept constant through rescaling of the atomic velocities every 100 fs. Unless stated, all simulations were performed within the NVT ensemble, and the temperature was kept constant by rescaling the atomic velocities.

The SCSP procedure consisted of four steps: (i) Prediction of the 2D crystallinity: MD calculations of various molecular short‐living aggregates were performed on a HOPG surface at 700 K for at least 10 ns. MD structures were classified and selected according to the intermolecular interaction energy and subsequently minimized. Planar unit cells (a, b, γ parameters) were extracted from the lowest energy structures after energy minimization. (ii) Construction of a first monolayer template (ML): each planar unit cell extracted from aggregates was replicated to obtain a first complete monolayer to be placed on a flat surface. However, due to the incommensurability between the DCV and HOPG supercells impeding an adequate ML building on either the employed HOPG surface or a new one, which could cause a loss of the planar ML morphology, we built a “square mesh” surface made of equidistant dummy atoms commensurate with the ML supercell within a margin of 1 Å. The Lennard‐Jones parameters of such atoms were adjusted to reproduce a similar energetic landscape to the one of HOPG surface. The template was placed on top of the dummy surface and a relaxation at MD level at *T *= 700 K was further performed for 1 ns on the system. (iii) Prediction of the 3D on‐surface crystallinity: we employed two approaches to create a high number of different frames of molecules placed on top of ML1, either through MD simulations (as done for step i) or systematic scans of position and orientation for planar aggregates. Structural minima were ranked according to the sum of the interaction energy within ML1 and ML2 molecules. The most stable structures were used to calculate the full set of 3D crystalline parameters (a, b, c, α, β, γ), see Tables S6 and S9. (iv) Prediction of the 3D bulk crystallinity: a bulk supercell of at least 50 molecules was built, replicating the 3D unit cell obtained in iii), and it was first subjected to a NVT simulation step of 0.2 ns followed by an NPT equilibration of 1 ns. Temperature was kept constant at 150 K (value detected during the experimental X‐ray measurements of DCV‐iPr) while a Berendsen barostat (compressibility of 10^−5^ bar^−1^) was introduced to keep the pressure at 1 atm during the NPT simulation. The system was then minimized, and the bulk unit cells were extracted from the last frame.

#### 4.4.3 XRD Simulations

The power diffraction patterns were simulated using the copper ray 1.54 Å. 1D and 2D GIWAXS patterns were simulated as described in Ref. [33] with a gaussian FWHM of 10° to introduce crystallites disorder and a Lorentzian FWHM of 1° to simulate instrumental broadening.

### 4.5 Vapor Deposition Simulations

Thin film growth was simulated using the open‐source program NAMD for the vapor deposition of the two molecules on a flat HOPG surface. The same force field parameters from Section 4.3.2 were used for modeling HOPG and DCV atoms during the simulations.

For DCV‐iPr, we built a 2D monolayer template of 60 molecules replicating one molecule of the experimental crystalline unit cell (*a* = 8.699, *b* = 6.823, *c* = 17.532, α = 90.00, β = 90.82, γ = 90.00) along *a* and *c* vectors (10 and 6 times, respectively). The total area of the monolayer is 86.994 × 105.190 Å^2^. The orthorhombic unit cell of High Oriented Pyrolytic Graphite (HOPG) (*a* = 2.456, *b* = 4.254, *c* = 6.696) was replicated respectively 36 and 25 times along the *a* and *b* axes to get the minimum surface area (88.416 × 106.350 Å^2^) that would fully contain the DCV template layer. The graphite supercell was also replicated 2 times along *c* to obtain four carbon layers. This graphite surface was employed both for the deposition of DCV‐iPr and for all SCSP calculations. The 2D template supercell was then placed on top of the graphite surface and equilibrated for 5 ns. Volume was kept constant and atomic velocities were rescaled every 100 fs to maintain the temperature constant at 500 K. PBCs were applied along *x* and *y* axes (*a*, *b* graphite axes), while the orthogonal dimension was set at 400 Å to simulate the vacuum. The two underneath layers of graphite were kept frozen to their positions, together with one atom of one molecule of the DCV layer, to avoid the drift over the surface.

For DCV‐Me, the system was built using a morphologically different monolayer template obtained from the replica (10 x 6) of the crystalline parameters reported in Table 1, calculated with the SCSP approach. The total area of the layer is 85.298 × 102.598 Å^2^, and a new graphite surface was built accordingly. We replicated the HOPG unit cell (35 × 24 × 2), obtaining a total area of 85.960 × 102.598 Å^2^. The monolayer was placed on top of the graphite surface and the system was equilibrated at the same conditions used for DCV‐iPr.

Then, for both DCV‐iPr and DCV‐Me, a sequence of MD simulations was performed to simulate the deposition of each molecule on the template placed on top of the graphite surface. A new molecule was introduced in the system after each deposition step (every 3 ns) with a random orientation and position along the xy plane, while the initial height (*z* axis) was set at 40 Å above the highest atom of the system with an initial z velocity of −0.5 Å /ps. The same conditions of temperature, volume, and PBC employed for the template equilibration on the surface were used at this stage for each deposition step. A total number of 315 molecules were deposited for each system.

### 4.6 Steady‐State Optical Properties Simulations

#### 4.6.1 The Frenkel–Exciton Hamiltonian

To model the electronic states and optical properties of realistic thin‐film aggregate morphologies we employ a Frenkel‐exciton‐type Hamiltonian coupled to the nuclear degrees of freedom (DoFs) of the aggregate (Equation (1)). This Hamiltonian is based on the assumption that all nuclear coordinates (R) of the system can be separated in two categories using a partition applied in other works [19, 29, 63]. The first, is a set of stiff modes, pertaining to the internal high‐frequency modes of the molecule ($R_{\mathrm{hf}}$), and the second is a set of soft modes, $R_{\mathrm{lf}}$, represented by the flexible DoFs of the molecules together with all the remaining environmental modes. The $R_{\mathrm{lf}}$ collection is treated at the classical level using configurations of molecules sampled along molecular dynamics and including environmental effects using a microelectrostatic (ME) model as described below. While the high‐frequency set, $R_{\mathrm{hf}}$ **,** is reintroduced and handled with a quantum‐mechanical vibronic treatment (see also Section S4). This partition essentially invokes the adiabatic approximation and assumes that the $R_{\mathrm{lf}}$ coordinates are much slower than the stiff ones, which can therefore rearrange very quickly to any $\text{Δ}R_{\mathrm{lf}}$ displacement. The Hamiltonian can be formalized as:

(1) $$\hat{H}={\hat{H}}_{\mathrm{FE}}\left(R_{\mathrm{lf}}\left(t\right)\right)+{\hat{H}}_{\mathrm{FE}-N}\left(R_{\mathrm{hf}}\right)$$

Here, ${\hat{H}}_{\mathrm{FE}}(R_{\mathrm{lf}}(t))$, represents the electronic part of the Hamiltonian written on a diabatic basis of localized Frenkel exciton (FE) states $\left|e_{k}\right\rangle$, where electron and hole are on the same site, *k*. In practice, ${\hat{H}}_{\mathrm{FE}}$, is written as:

(2) $${\hat{H}}_{FE}^{\left(n\right)}\left(R_{\mathrm{lf}}\left(t\right)\right)=\sum_{k}^{M}\left(E_{S_{0}\to S_{1}}^{\text{film},k}\left(R_{\mathrm{lf}}\left(t\right)\right)\right)\left|e_{k}\right\rangle \left\langle e_{k}\right|+\sum_{k,l\neq k}^{M}V_{kl}\left(R_{\mathrm{lf}}\left(t\right)\right)\left|e_{k}\right\rangle \left\langle e_{l}\right|$$

${\hat{H}}_{\mathrm{FE}}(R_{\mathrm{lf}}(t))$ depends on the actual configurations of the molecules in their thin‐film morphology and their dynamics over time (namely, this Hamiltonian can be reconstructed for each MD configurations, *n*, along time (*t*)). The time‐dependence of $R_{\mathrm{lf}}(t)$ indicates the fact that low‐frequency vibrations can change along the different snapshots of the MD (as detailed in Section S9). The diagonal elements are the excitation energies $E_{S_{0}\to S_{1}}^{\text{film},k}(R_{\mathrm{lf}}(t))$ associated to the localized molecular excitation of each molecule k embedded and polarized by the other molecules within the thin‐film aggregate, while the off‐diagonal are the long‐range excitonic interactions ($V_{kl}(R_{\mathrm{lf}}(t))$) between tightly bound excitons sitting either on molecules k or l.

The vibrational motion due to stiff high‐frequency modes enters the second part of the Hamiltonian ${\hat{H}}_{\mathrm{FE}-N}(R_{\mathrm{hf}})$, in Equation (1). Since the coordinates $R_{\mathrm{hf}}$ instantaneously rearrange to the new configuration of $R_{\mathrm{lf}}(t)$, do not depend on the time. These Cartesian coordinates are converted in normal modes and the coupling between the excitonic wavefunction and the quantized high‐frequency modes is written using a displaced (quantum) harmonic oscillator model as described in Sections S4, S5. To make the problem computationally tractable all intramolecular high‐frequency vibrations are grouped into a single effective mode as often done in the literature [17, 18]. The effective Huang–Rhys factor $\left(S_{\mathrm{eff}}=\frac{\lambda_{\mathrm{hf}}^{\mathrm{rel}}}{\hbar \omega_{\mathrm{eff}}}\right)$ and the frequency, $\hbar \omega_{\mathrm{eff}}$, of such an effective mode are used to construct the displaced excited state harmonic potential and to obtain the Franck–Condon integrals. The values are calculated as described in Sections S4, S5. The Hamiltonian becomes:

(3) $${\hat{H}}_{\mathrm{FE}-N}=\hbar \omega_{\mathrm{eff}}\sum_{k}^{M}b_{k}^{†}b_{k}+\hbar \omega_{\mathrm{eff}}\sqrt{S_{\mathrm{eff}}}\sum_{k}^{M}\left(b_{k}^{†}+b_{k}+\sqrt{S_{\mathrm{eff}}}\right)\left|e_{k}\right\rangle \left\langle e_{k}\right|$$

where $b_{k}^{†}$ and $b_{k}$ are the common creation and annihilation operators associated with a quantum harmonic oscillator. Equation (3) assumes that both excited and ground states have the same curvature and zero‐point energy, which is uniform for all molecules.

To summarize, the Frenkel–exciton Hamiltonian (in Equation (1)) contains three important ingredients that are computed from first principles TDDFT: excitation energies, excitonic couplings and electron‐vibration couplings. All these ingredients needed for the parametrization were computed at TDDFT level using the ωB97X‐D/6‐31G(d, p), unless otherwise stated. Once constructed, the Hamiltonian can be diagonalized to compute optical absorption and emission of extended aggregates with Equations S4, S12. Different versions of such a Hamiltonian have been used with success to model optical [17, 18] as well as exciton transport properties [37, 64, 65] of various application‐relevant opto‐electronic materials. In this work, it was used to describe the excitonic states of both deposited thin‐film morphologies produced by vapor deposition simulations as described above (see Figure 5), as well as crystalline samples (Figure S18) of both DCV‐iPr and DCV‐Me systems.

#### 4.6.2 Excitonic Interactions

The excitonic couplings, $V_{kl}(R_{\mathrm{lf}}(t))$, are calculated here using either an accurate diabatization scheme—the multi‐state fragment charge‐difference fragment excitation‐difference method (MS‐FED‐FCD) [35, 36, 66]—on a few selected pairs, or a simplified approach based on a Coulomb sum between transition densities allowing for the simulation of realistic morphologies and system sizes. The transition densities are approximated here using atom‐centered transition charges using the transition electrostatic potential charges (TrESP) approach [67]. The TrESP charges were obtained as proposed by Renger et al. in Ref [67], by fitting the electrostatic potential generated by the transition density at the optimized geometry of the molecule in vacuo. TrESP charges are used to compute all the long‐range excitonic interactions in the Hamiltonian in Equation (2) in a very fast analytic manner. Since these interactions are calculated between molecules in vacuo, the effect of the environment on the interactions is taken into account with an isotropic screening with dielectric constant (ε = 3) [29, 68].

As discussed in Section S10, the TrESP method not only provides a fast and reasonably precise way of computing all the electronic couplings between k and l molecules but also ensures consistency in the signs of the couplings across different molecular pairs and across MD snapshots. The significance of this method for calculating excitonic couplings has been discussed in previous works [19, 29, 37, 66]. We highlight that the off‐diagonal disorder associated with (slow) fluctuations of the excitonic couplings, $V_{kl}(R_{\mathrm{lf}}(t))$, in the deposited thin‐film structures (in Figure 4) is accounted for here by evaluating these interactions between all pairs extracted for a set of 100 snapshots extracted from 0.5 ns long MD trajectories. This is only possible owing to the fast TrESP method that allows us to compute a very large number of couplings for different structures. No off‐diagonal disorder is included in the simulation of the crystalline sample instead.

#### 4.6.3 Excitation Energies

The excitation energies $E_{S_{0}\to S_{1}}^{\text{film},k}(R(t))$ in general depend on the Cartesian coordinates R(t) of the molecule k, at a given time. Noting that the high‐frequency part is already considered in the vibronic part of the Hamiltonian (Equation (3)), the excitation energies are calculated for each molecule in the sample as a function of low‐frequency displacements, as 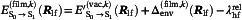. Here, 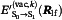 is the vertical excitation calculated at TDDFT level of theory for the molecule *k* extracted in vacuo. The starting structures were appositely minimized at MM level, introducing high force constants for the bonding terms of our FF. This was done to avoid high‐frequency modes (already treated at quantum level) to also contribute to the diagonal energetic disorder. ${\text{Δ}}_{\mathrm{env}}^{\left(\text{film},k\right)}(R_{\mathrm{lf}})$ is the environmental energy calculated with the classical ME model to account for electrostatic and induce‐dipoles effect associated with the neighboring molecules present in the thin‐film [41, 42]. Finally, $\lambda_{\mathrm{hf}}^{\mathrm{rel}}$ is the exciton relaxation energy due to the high‐frequency modes that need to be subtracted to the vertical excitation to find the adiabatic excitation energy of the molecules in the thin‐film phase. When a disordered morphology is considered, the positional static disorder characterizing such a system is readily accounted as the standard deviation of $E_{S_{0}\to S_{1}}^{\left(\text{film},k\right)}(R_{\mathrm{lf}})$, which is different for each molecule in the disordered sample. When we consider a perfect crystalline sample, $E_{S_{0}\to S_{1}}^{\left(\text{crys},k\right)}(R_{\mathrm{lf}})$ is assumed to be the same for all the molecules (Figure S18).

The conformational disorder of a molecule along dynamics is evaluated through the autocorrelation function of the vertical excitation energies 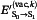 (Equation S7) and the subsequent cosine transformation of energy fluctuations (Equation S9). This energy was computed for each snapshot every 10 fs along a 20‐ps‐long MD run. Notably, a clear distinction emerges between the contributions of stiff high‐frequency and flexible low‐frequency vibrations to the energy distribution (see Figure S17), validating the partition discussed above. The high‐frequency vibrations, $R_{\mathrm{hf}}$ (modes > 1100 cm^−1^), associated with molecular stretching and ring‐breathing motions, are incorporated into the vibronic part of the Hamiltonian (Equation (3)) through an effective high‐frequency mode. This dynamic contribution to the total conformational disorder is introduced by further broadening (i.e., randomly sampling them from a Gaussian distribution) the excitation energies containing a degree of positional disorder. This allows us to capture the total conformational disorder $\sigma_{\text{conf}}$ (see Table 3) as formed by static and dynamic contributions.

The environmental effect was accounted for using the classical microelectrostatic (ME) model with the induced‐dipole model implemented in the MESCal code [41, 42]. The ME model was parametrized by computing gas‐phase electrostatic potential (ESP) atomic charges with ωB97X‐D/6‐31G++(d, p) level of theory. ESP charges were obtained for all neutral and excitonic S1 states of the individual molecules of the thin‐film sample extracted from the last MD frame of a 0.5‐ns‐long NVT trajectory. The polarizability tensor of the neutral species computed at the same level of theory as above was applied to describe both the neutral and the excitonic states. In order to evaluate electrostatic interactions, self‐consistent calculations were performed on the thin‐film sample, where PBCs were applied in the x–y plane with a large cutoff of 800 Å to ensure convergence of the electrostatic energies. The induction term was evaluated separately on spherical clusters where each molecule in the thin film was placed at the center of the cluster.

## Supporting Information

Additional supporting information can be found online in the Supporting Information section. Details on photovoltaic responses, evaluation of molecular stability, calculations of molecular excitation energies, relaxation energies and Huang‐Rhys factors, calculations of optical molecular spectra, further details on crystal structure predictions, further details on thin‐film structures, Hamiltonian and coupling matrix elements, spectral density and dynamic conformational disorder, further details on calculated solid‐state spectra, further experimental spectra. A .cif file of DCV‐iPr single crystal data can be obtained free of charge from The Cambridge Crystallographic Data Centre via www.ccdc.cam.ac.uk/data_request/cif under entry number 2392619.

## Conflicts of Interest

The authors declare no conflicts of interest.

## Supporting information

Supplementary Material

## Data Availability

The data that support the findings of this study are available from the corresponding authors upon reasonable request.
