# Supplementary material for: On the Design of Steep Optical Absorbers for Vacuum‐Processed Organic Solar Cells: One Isopropyl Group Makes the Difference
Source: Small Sci. 2026 May 15;6(5):e202500633. doi: 10.1002/smsc.202500633 (PMC13179045; doi:10.1002/smsc.202500633)
Supplement: Supplementary file 1 — Supplementary Material [file SMSC-6-e202500633-s001.pdf]

Supplementary Information:

On the design of steep optical absorbers for vacuum-processed organic solar cells: One isopropyl group makes the difference

Mohamed El habib Bouajhine[a]‡, Emilio Lorini[b]‡, Samuele Giannini[c]‡,* Siebe Frederix[d,e], Karsten Walzer[f], Marieta Levichkova[f], Gunter Mattersteig[f], Martin Pfeiffer[f], Eva Bittrich[g], Petra Uhlmann[g], Vincent Lemaur[a], Koen Vandewal[d,e], Patrick Brocorens[a], Luca Muccioli[b], David Beljonne[a,*]

a) Laboratory for Chemistry of Novel Materials, University of Mons, Mons 7000, Belgium
b) Department of Industrial Chemistry, University of Bologna, Via Gobetti 85, 40129 Bologna, Italy
c) Department of Chemistry and Industrial Chemistry, University of Pisa, via G. Moruzzi, 13, 56124 Pisa, Italy
d) Hasselt University, imec, Institute for Materials Research (imo-imomec), Wetenschapspark 1, B-3590 Diepenbeek, Belgium
e) Energyville, imo-imomec, Thorpark 8320, B-3600 Genk, Belgium
f) Heliatek GmbH, Treidlerstrasse 3, 01139 Dresden, Germany
g) Leibniz-Institut für Polymerforschung Dresden e.V., Dresden 01069, Germany

‡ These authors contributed equally to this work.

Section S1: Photovoltaic response

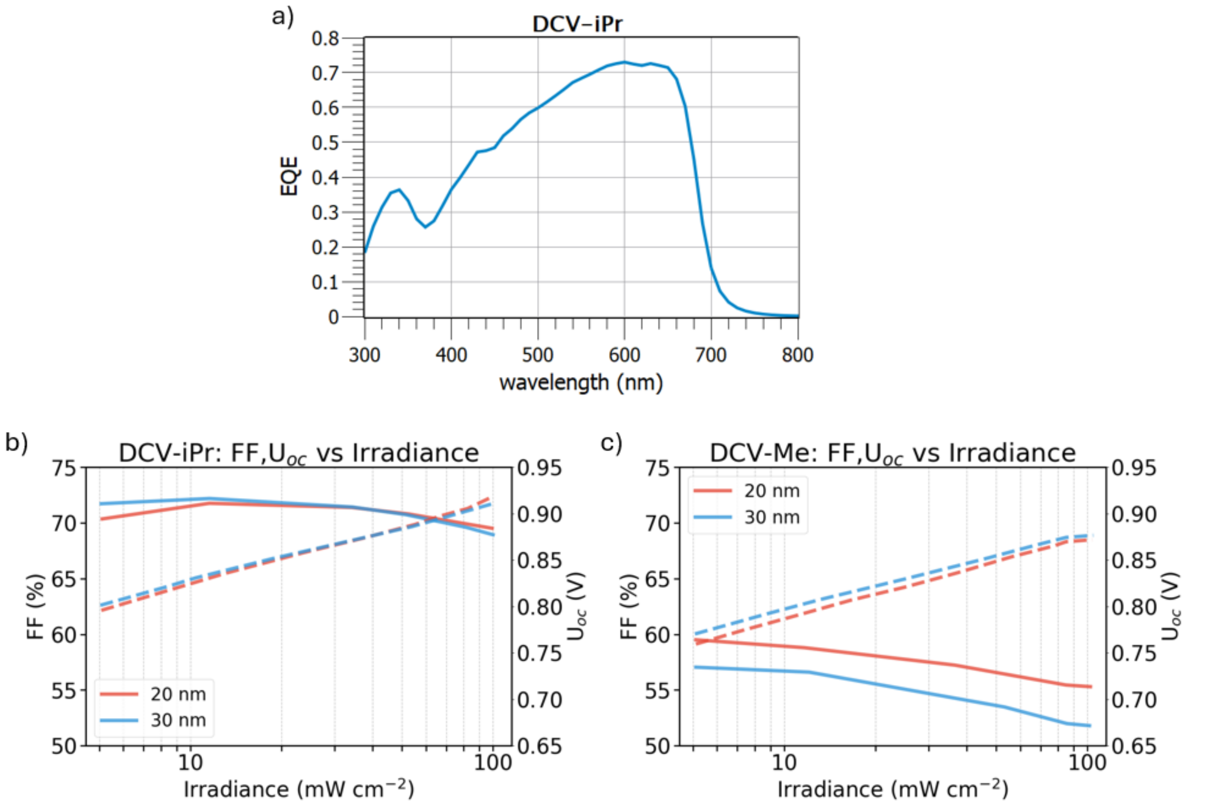

Figure S1: Photovoltaic responses of DCV-iPr:C_{60} (2:1, 50°C) and DCV-Me:C_{60} (1:1, RT) devices. Panel a) represents the EQE spectrum of DCV-iPr:C_{60} with 30nm active layer. Panels b) and c) show the dependence of FF and V_{oc} on the irradiance for DCV-iPr:C_{60} and DCV-Me:C_{60} with different blend layer thicknesses, respectively.

Section S2: Molecular stability

Below, we investigate the stability of the different conformers of both DCV derivatives as isolated molecules. All the potential minima of the molecule are generated by systematically modifying all the relevant torsion angles (Fig. S2) and optimizing the resulting geometries by molecular mechanics using a quantum-mechanically parametrized FF as explained in the Methods in the main text.

DCV-iPr molecule

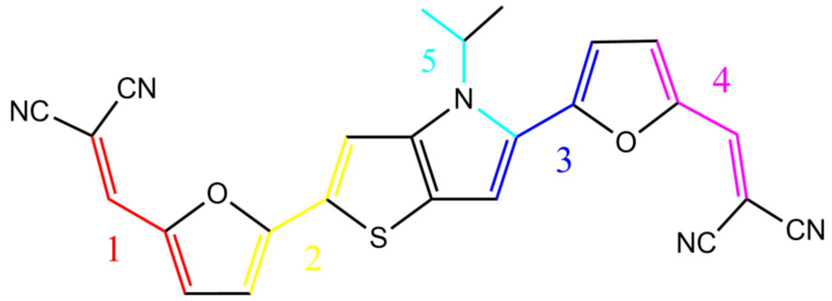

Figure S2: DCV-iPr molecule with the numbering of the torsional angles that were modified during the conformational search.

For DCV-iPr, 700 conformations were generated through a systematic conformational search by varying the five torsion angles. Their optimized torsion angles are shown in Fig. S3. After removing redundant conformations, 56 local energy minima remained. Their optimized torsion angles and relative energies calculated with the Dreiding force field, are listed in Table S1.

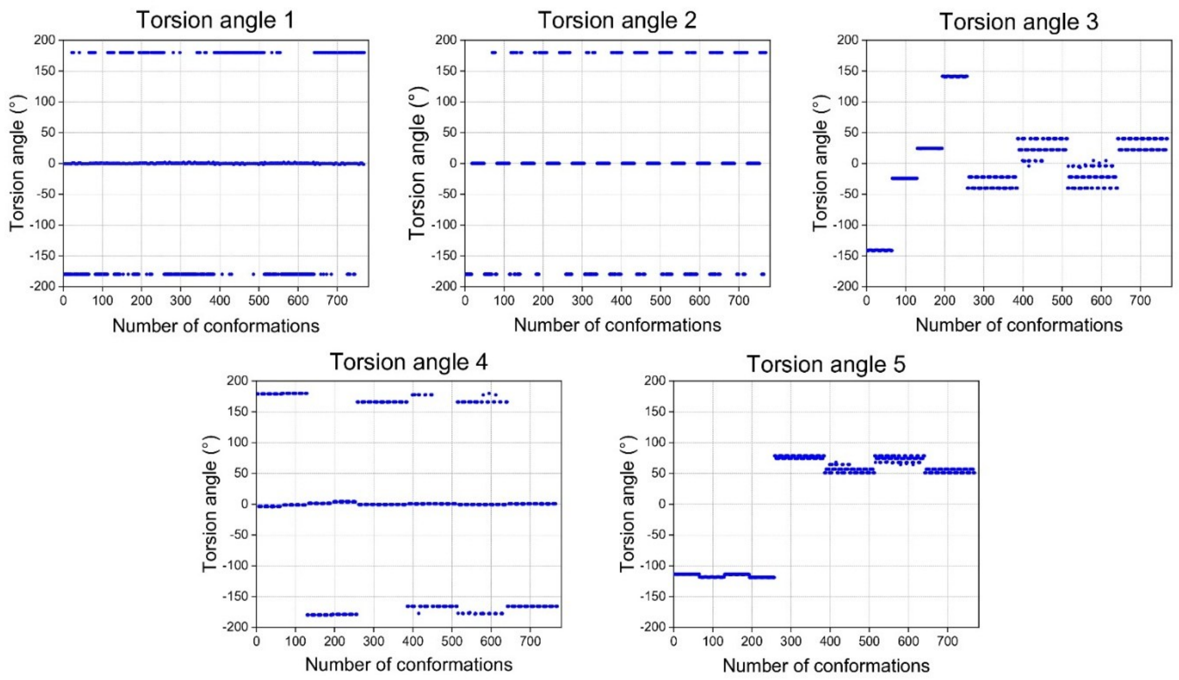

Figure S3: Torsion angle values obtained during the conformational search of DCV-iPr, using the Dreiding force field

Table S1: Energetic classification (in kcal/mol) of the DCV-iPr conformations in vacuo.

Conformer	Torsion1	Torsion2	Torsion3	Torsion4	Torsion5	Relative energy
1	-179.99	179.99	24.28	-179.81	-114.20	0.00
2	179.98	-179.99	-24.28	179.81	-118.29	0.00
3	-179.98	0.01	-24.33	179.86	-118.25	0.53
4	179.98	-0.01	24.34	-179.88	-114.19	0.53
5	-179.99	-179.98	-141.48	178.99	-113.61	1.89
6	179.99	179.98	141.49	-178.99	-119.00	1.89
7	179.99	179.99	3.90	177.69	64.50	2.51
8	-180.00	-179.99	-3.86	-177.71	67.64	2.51
9	-179.99	0.08	-141.40	179.02	-113.62	2.52
10	179.99	-0.09	141.40	-179.02	-118.94	2.52
11	0.08	-179.99	-24.38	179.83	-118.28	2.63
12	-0.08	179.99	24.38	-179.84	-114.19	2.63
13	179.95	179.90	-24.20	-1.30	-118.69	2.93
14	-179.95	-179.90	24.20	1.31	-113.76	2.93
15	180.00	0.00	3.96	177.66	64.49	2.94
16	-179.98	0.05	-5.21	-176.91	68.17	2.94
17	-0.37	0.10	24.45	-179.84	-114.18	2.97
18	0.36	-0.10	-24.45	179.84	-118.26	2.97
19	179.99	179.95	40.18	-165.84	50.98	3.20
20	-179.98	-179.94	-40.18	165.84	78.42	3.20
21	179.99	0.12	24.37	1.25	-113.71	3.50
22	-179.99	-0.12	-24.37	-1.25	-118.69	3.50
23	179.99	-0.06	40.14	-165.93	51.04	3.63
24	-179.98	0.06	-40.14	165.93	78.35	3.63
25	-179.97	-179.95	-140.77	-3.59	-113.72	4.29
26	179.97	179.95	140.77	3.60	-118.90	4.29
27	-0.39	-179.94	-141.44	179.01	-113.64	4.56
28	0.39	179.94	141.44	-179.01	-118.95	4.56
29	-179.98	-179.99	-22.00	-0.64	74.35	4.61
30	179.98	179.98	22.01	0.62	56.74	4.61
31	180.00	-0.07	140.68	4.18	-118.85	4.89
32	179.99	0.08	-140.69	-4.18	-113.71	4.89
33	0.50	-0.10	141.37	-179.06	-118.95	4.92
34	-0.51	0.10	-141.37	179.07	-113.61	4.92
35	0.07	180.00	-4.58	-177.29	67.92	5.06
36	-0.08	-180.00	4.58	177.29	64.23	5.06
37	-180.00	-0.06	-22.21	-0.53	74.42	5.11
38	-179.99	0.06	22.15	0.37	56.69	5.11
39	0.05	-0.02	-4.50	-177.34	67.89	5.41

40	-0.05	0.02	4.50	177.34	64.26	5.41
41	-0.10	179.95	-24.31	-1.34	-118.68	5.58
42	0.09	-179.95	24.31	1.34	-113.74	5.58
43	-1.39	179.92	40.12	-165.82	51.03	5.74
44	1.40	-179.92	-40.12	165.82	78.37	5.74
45	0.07	-0.06	-24.25	-1.02	-118.66	5.94
46	-0.07	0.06	24.25	1.02	-113.74	5.94
47	0.25	-0.09	-40.31	165.88	78.41	6.08
48	-0.25	0.08	40.32	-165.88	50.98	6.08
49	-0.41	-179.98	-140.75	-4.01	-113.72	6.97
50	0.41	179.98	140.75	4.02	-118.87	6.97
51	0.20	-179.97	22.11	0.73	56.72	7.19
52	-0.20	179.97	-22.11	-0.73	74.36	7.19
53	0.52	0.08	-140.75	-3.18	-113.72	7.32
54	-0.53	-0.08	140.75	3.18	-118.85	7.32
55	-0.13	0.00	22.08	0.43	56.73	7.55
56	0.13	0.00	-22.08	-0.43	74.35	7.55

In all conformations, the isopropyl group is out-of-plane, as indicated by the values of torsion angle 5 (refer to Fig. S2). For the backbone, torsion angles 1, 2, and 4 adopt either 0° or 180°, while angle 3 can take several values: 0°, 25° (or -25°), 40° (or -40°), and 140° (or -140°). The most stable conformation has angle 3 at 24°, and the molecule adopts a C-shaped geometry, where the two cyano groups are oriented in the same direction (Figure S4). However, the conformation found in the crystal structure (i.e., conformation n°15) has angle 3 at 4°, resulting in an almost fully planar backbone.

Only a few conformations exhibit a planar angle 3, and they are not the most stable ones due to steric hindrance with the neighboring isopropyl group. However, their planarity likely promotes close packing in the crystal, leading to an energy gain compared to conformations with distorted backbones. Although an intramolecular energy criterion (molecules in vacuo) can help to identify initial candidates for crystallization, the introduction of intermolecular interactions in the solid state can alter the stability ranking. In this case, the experimentally observed conformation (n°15) appears relatively high in the ranking, with an energy difference of 2.94 kcal/mol above the global minimum. For DCV-Me, we thus systematically tested all the generated conformations to build polymorphs.

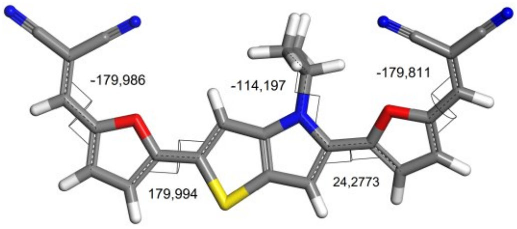

Figure S4: the most stable conformation of isolated DCV-iPr optimized by MM (which has a C-shape).

DCV-Me molecule

For DCV-Me, 16 local energy minima were identified through a systematic conformational search by varying the four torsion angles shown in Fig S5. Their optimized torsion angles and relative energies, calculated using the Dreiding force field, are listed in Table S2. Since the bulky isopropyl group in DCV-iPr has been replaced by a methyl group in DCV-Me, steric effects at torsion 3 are reduced. As a result, all four backbone angles adopt either 0° or 180°, meaning that all conformations are fully planar.

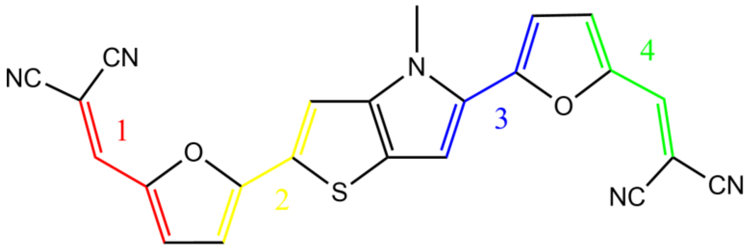

Figure S5: DCV-Me molecule with the numbering of the torsional angles that were modified during the conformational search.

Table S2: Energetic classification (in kcal/mol) of the DCV-Me conformations in vacuo.

	**Torsion 1**	**Torsion 2**	**Torsion 3**	**Torsion 4**	**Relative energy**
Conformation 1	-179.59	-179.40	0.09	179.71	0.00
Conformation 2	-4.01	-179.62	0.56	179.73	1.26
Conformation 3	179.99	-0.25	0.07	179.02	1.57
Conformation 4	178.35	178.43	-2.12	-6.10	2.08
Conformation 5	-179.79	179.66	-178.42	-179.73	2.32
Conformation 6	-3.49	0.84	0.88	179.85	2.81
Conformation 7	1.36	180.00	0.05	-0.90	4.26
Conformation 8	-0.93	-179.55	179.74	-179.15	4.46
Conformation 9	-179.83	-179.77	-178.41	-1.41	4.52
Conformation 10	-179.98	1.04	-0.26	-2.88	4.70
Conformation 11	-178.76	2.43	-172.96	179.00	5.35
Conformation 12	-5.13	1.70	0.78	-2.90	6.03
Conformation 13	-1.82	0.80	179.53	179.05	6.37
Conformation 14	-0.20	-179.69	-177.25	-0.43	6.71
Conformation 15	-179.47	0.28	-179.14	0.26	7.24
Conformation 16	-0.86	0.13	177.91	-0.88	8.25

These conformations were used to generate polymorphs, whose relative energies, calculated using the Dreiding force field and DFT-D, are presented in Table S5, respectively. The most stable conformation adopts a planar C-shaped geometry (Fig S6). However, it is the third lowest-energy conformation, with an S-shape, that leads to the polymorph with the lowest energy.

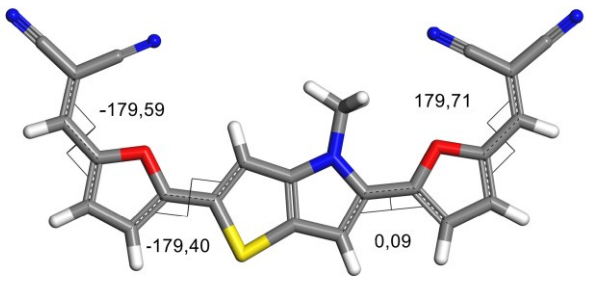

Figure S6: Global minimum of DCV-Me in vacuo (with a C-shape).

Section S3: Excitation energies

As shown above, the isolated DCV derivatives in the C-shape and S-shape configurations exhibit similar stability in vacuo. However, as we discuss below, the S-shape configuration is the one actually observed in the solid-state morphology of both DCV-Me and DCV-iPr. We verify below that, besides similar ground state energies, both conformers exhibit similar excited-state properties.

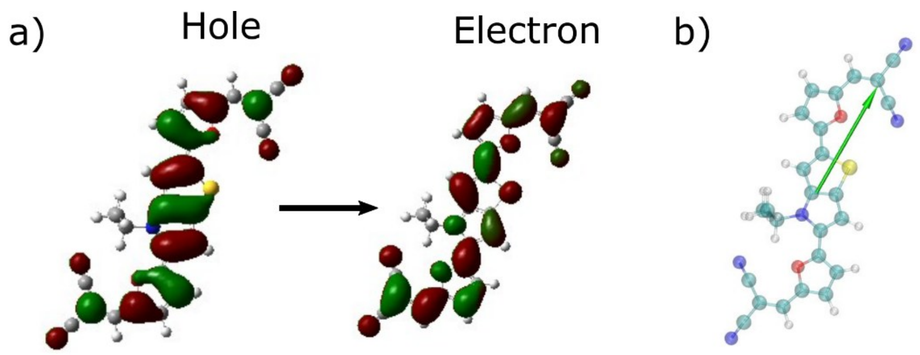

Figure S7: a) Natural transition orbitals (NTOs) for the first singlet excited state at ωB97X-D /6-31G(d,p) level of theory for DCV-iPr molecule. b) Transition dipole moment corresponding to the transition density represented in a). Note that there is not density on the isopropyl group, so the NTOs and transition dipole direction is the same for DCV-Me as well.

Table S3: Excitation energy (eV) and oscillator strength (*f*) for the lowest-energy (singlet) states using ωB97X-D level of theory for DCV-iPr and DCV-Me molecules. Basis set fixed at 6-31G(d,p) level.

	DCV-iPr		DCV-Me	
	energy [eV]	f	energy [eV]	f
Opt S-shape (vacuo)	2.739	1.689	2.764	1.713
Opt S-shape (DMAc)[a]	2.588	1.906	2.602	1.912
Opt C-shape (vacuo)	2.731	1.055	-	-
Opt C-shape (DMAc)[a]	2.597	1.188	-	-

[a] The solvent has been added using a Polarizable Continuum Model with dielectric constants characteristic of Dimethylacetamide solvent.[1]

Section S4: Relaxation energies

The molecular reorganization energies associated with the excitation of the DCV-molecules were evaluated using the 4-point approach described, *e.g.*, in Ref.[2–4]:

$$\lambda^{4-\text{point}} = \lambda_{EX}^{\text{rel}} + \lambda_{N}^{\text{rel}} = [E_{EX}(\boldsymbol{R}_N) + E_N(\boldsymbol{R}_{EX})] - [E_{EX}(\boldsymbol{R}_{EX}) + E_N(\boldsymbol{R}_N)] \quad \text{(S1)}$$

where $\lambda_{EX}^{\text{rel}}$ and λ_{N}^{rel} are the relaxation energies of the excited and neutral states, respectively. $E_{EX(N)}(\boldsymbol{R}_{N(EX)})$ is the energy of the excited (neutral) molecule in the optimized neutral (excited) state and $E_{EX(N)}(\boldsymbol{R}_{EX(N)})$ is the energy of excited (neutral) molecule in the optimized excited (neutral) minimum. We found $\lambda^{4-\text{point}}$ = 0.327 eV and 0.332 eV DCV-iPr and DCV-Me, respectively. These values were computed using ωB97X-D/6-31G(d,p) level of theory. The fact that the reorganization energies of the two molecules are similar confirm the fact that the ground and excited state potentials are comparable for both systems.

Section S5: Huang-Rhys factors

A complementary useful approach to calculate the relaxation energies of excited and ground states and to determine the parameters required to construct the Frenkel Hamiltonian in Eq. 1 of the main text (namely, the Huang-Rhys factor and effective frequency) involves their decomposition into contributions relative to each normal mode. This method is herein referred to as normal mode analysis (NMA). The total relaxation energy of the excited and neutral state can be written as a sum of the individual contributions (see Fig. S8):

$$\lambda_{N(EX)}^{\text{rel}} \cong \sum_i \hbar\omega_i S_i \quad \text{(S2)}$$

where S_i denotes the Huang-Rhys factor of a specific mode of frequency $\hbar\omega_i$ of the normal modes of the ground or excited state, respectively. The values of reorganization energies and Huang-Rhys factor are reported in Figure S8 and Table S4. To obtain these values, we employed FCclasses3.0 code,[5] and used the Adiabatic Hessian (AH) approach in which the final PES is expanded around its own equilibrium structure found after excited state optimization. The calculated gradient and Hessian matrix at the minimum of the excited state potential allow for a reconstruction of the excited state potential energy surface by taking into account Duschinsky's normal mode transformation.[6]

Table S4: Relaxation energies (in eV) and Frenkel Exciton Hamiltonian parameters for S-shape and C-shape configurations.

	DCV-iPr		DCV-Me
	S-shape	C-shape	S-shape
λ^{rel}	0.164	0.175	0.167
$\lambda_{\text{hf}}^{\text{rel}}$	0.123	0.125	0.126
$\lambda_{\text{lf}}^{\text{rel}}$	0.041	0.050	0.040

$\hbar\omega_{\rm eff}$	0.190	0.191	0.191
$S_{\rm eff}$	0.646	0.656	0.663
$\sigma^{\rm hom.}$	0.046	0.051	0.046

In Fig. S8, we show also that the relaxation energies of both DCV-iPr and DCV-Me is similar and, in both molecules, there are a few modes that are mostly coupled with the excitation involving vibrational frequencies that are essentially located above 1100 cm-1. Thus, in a first approximation, as done in other works,[7] we assumed that part of the total relaxation energy associated to the formation of an excited state is carried out by a single high-frequency mode with an effective frequency $\hbar\omega_{\rm eff}$. This quantity was evaluated for each excitation by weighting the frequencies of each mode across the spectrum above 1100 cm-1 in Fig. S8 by the corresponding Huang-Rhys factor S_i, namely $\hbar\omega_{\rm eff} = \sum_i \hbar\omega_i S_i / \sum_i S_i$. The effective Huang-Rhys factor associated to the high-frequency relaxation energy ($\lambda_{\rm hf}^{\rm rel}$) becomes $S_{\rm eff} = \lambda_{\rm hf}^{\rm rel} / \hbar\omega_{\rm eff}$. A summary of the parameters used to build the Hamiltonian in Eq. 1 for solid-state aggregates is reported in Table S4. The rest of the relaxation energy associated with low-frequency modes ($\lambda_{lf}^{\rm rel}$) was assumed to be classical in nature and, thus, to contribute to the homogeneous broadening of the spectrum ($\sigma^{\rm hom.} = \sqrt{2K_B T \lambda_{\rm lf}^{\rm rel}}$).

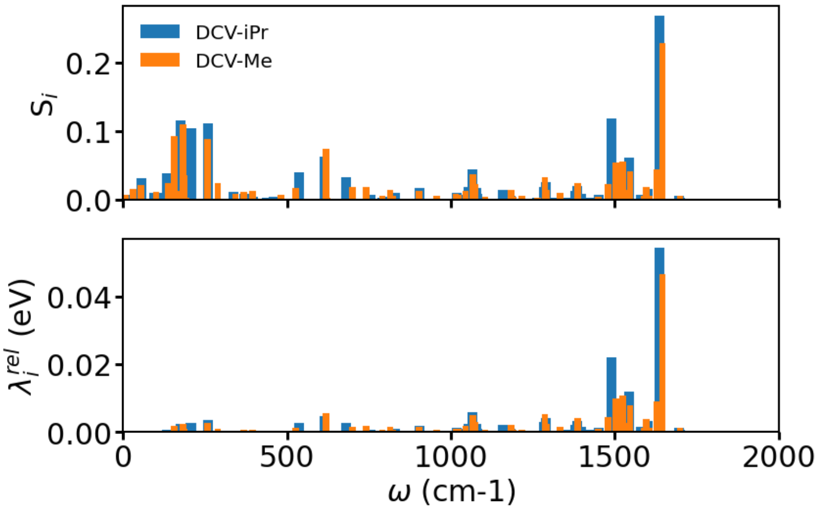

Figure S8: Huang-Rhys factors (top panels) and relaxation energies (bottom panels) when going from the neutral to the excited state geometry. The analysis was performed using the Adiabatic Hessian (AH) models by employing with FCclasses3.0.[5]

Section S6: Molecular spectra

The absorption spectrum is defined as:

$$A(E) = \frac{1}{|\mu|^2} \sum_j E_j \left| \langle \Psi^{(g)} | \hat{\mu} | \Psi^{(j)} \rangle \right|^2 W(E - E_j) \quad \text{(S3)}$$

where $\hat{\mu}$ is the transition dipole moment operator between the ground state wavefunction $\Psi^{(g)}$ and a given eigenstate $\Psi^{(j)}$ of the system, and W is a line broadening function which in this work is taken to be a Gaussian function with a standard deviation σ. The line intensity is the oscillator strength calculated as the product of the square of the transition dipole moment and the transition energy (E_j).

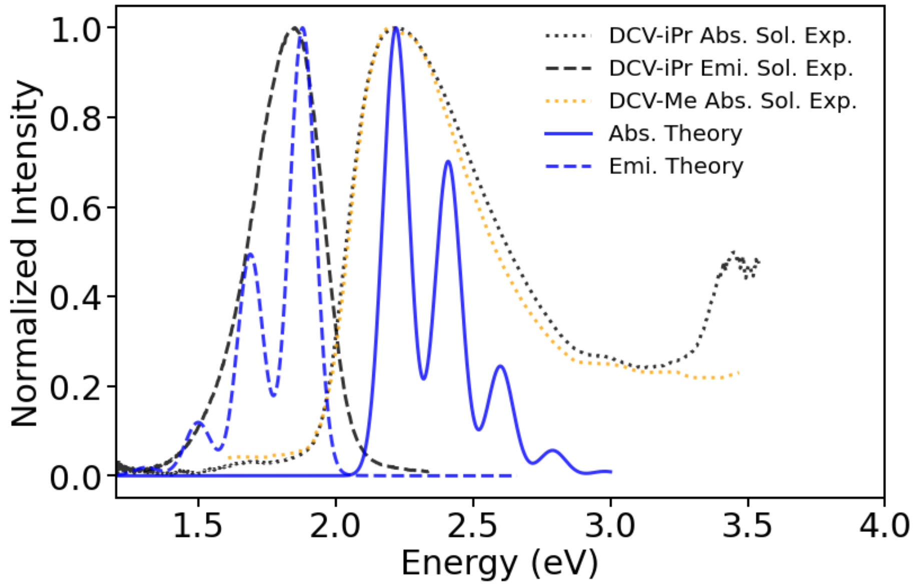

Figure S9: Single molecules spectra (theory vs experiment). Experimental absorption and emission spectra for DCV-iPr and DCV-Me molecules in DMAc solution. Experimental details are in Methods (in the main text). The theoretical spectra were computed using a single effective mode considering the parameters in Table S4 related to ωB97X-D /6-31G(d,p) level of theory, by considering a single-effective mode coupled to the excitation as discussed in the text. The computed absorption and emission spectra were broadened using a $\sigma^{\text{hom.}}$ =46 meV and shifted by 0.54 eV to correct for the TD-DFT error. Note that the Stokes shift of 328 meV, is related to the molecular relaxation and comes directly out from the simulation considering the total reorganization energy.

For a single molecule the spectrum is computed using the Franck-Condon (FC) approximation which permits to write $\left|\langle\Psi^{(g)}|\hat{\mu}|\Psi^{(j)}\rangle\right|^2 = |\mu|^2 \exp(-S_{\text{eff}}) \frac{S_{\text{eff}}^{\tilde{\nu}}}{\tilde{\nu}!}$. When considering only one electronic transition ($S_0 \rightarrow S_1$), and one effective vibrational mode connected to the excitation the line shape of the absorption spectrum takes the form:

$$A(\omega) = |\mu|^2 \exp(-S_{\text{eff}}) \sum_{\tilde{\nu}=0}^{\tilde{\nu}_{max}} \frac{S_{\text{eff}}^{\tilde{\nu}}}{\tilde{\nu}!} \exp\left[-\frac{\left(E - \left(E_{S_0 \rightarrow S_1} + \tilde{\nu}\hbar\omega_{\text{eff}}\right)\right)^2}{2\sigma^2}\right] \quad \text{(S4)}$$

where $E_{S_0 \rightarrow S_1}$ is the $S_0 \rightarrow S_1$ electronic transition energy, $\tilde{\nu} = 0,1,2\ldots$, denotes the number of vibrational energy levels of the excited states spaced by an effective vibrational frequency $\hbar\omega_{\text{eff}}$. S_{eff} is the Huang Rhys factor of the electronic transition, which was calculated as previously described. To mimic the effect of the environment and related homogenous spectral broadening, a Gaussian line shape with constant standard deviation ($\sigma^{\text{hom.}}$) was assumed for all overtones. The comparison between theory and experiments resulting from this analysis is reported in Fig. S9.

Section S7: Further details on crystal structure predictions

The identification of the single crystal involves selecting, among all the polymorphs generated in the five space groups and the representative conformations (for DCV-Me), the crystal structure with the lowest energy and highest density. Initially, the energy ranking is determined using the adapted Dreiding force field. To refine this ranking, we further optimized the geometries of the polymorphs using DFT-D (where D stands for dispersion) and classified the polymorphs by their DFT-D energies as described in Methods.

DCV-iPr crystal

In Table S5, we notice that the relative energies obtained from the force field do not accurately reflect the true stability of the structures. Indeed, the most stable structure should belong to the $P2_1$ space group (as observed experimentally) rather than P-1. Using DFT-D, however, as expected, the most stable structure corresponds to the $P2_1$ space group.

Table S5: Relative energies (in kcal/mol) of the most stable DCV-iPr structures obtained using the force field and further optimized with DFT-D.

Relative energy	$\boldsymbol{P_{21/c}}$	$\boldsymbol{P_{-1}}$	$\boldsymbol{P_{212121}}$	$\boldsymbol{C_{2/c}}$	$\boldsymbol{P_{21}}$
Force field	0.0	0.0	0.4	1.8	0.9
DFT-D optimization	2.2	1.5	2.0	4.7	0.0

In Table S6, the experimental crystalline parameters of DCV-iPr single crystals are reported alongside the calculated parameters for BCSP and SCSP. For SCSP, the unit cell extracted in the presence of the HOPG surface (step (iii) of section 4.3.2) is shown to converge toward values similar to those of the experimental and BCSP unit cells after the surface is removed and 3D periodic boundary conditions are applied (step (iv)).

Table S6: Lattice lengths (Å) and angles (°) of the polymorph obtained by on-surface modelling (SCSP on-surface), then further optimized with FF without influence of the substrate (SCSP bulk), and the polymorph obtained by bulk modelling (BCSP) of DCV-iPr.

		a	*b*	*c*	*α*	*β*	*γ*	*Z*
Exp		8.7	6.8	17.5	90.0	90.8	90.0	2
SCSP	*On-surface*	8.8	7.3	17.2	101.1	95.6	90.3	2
	Bulk	8.7	6.9	17.5	89.1	88.5	88.7	2
BCSP		8.7	6.9	17.5	90.0	89.8	90.0	2

DCV-Me crystal

Table S7: Relative energies (in kcal/mol) of the DCV-Me structures optimized with the force field.

**relative energy**	$\boldsymbol{C_{2/c}}$	$\boldsymbol{P_{-1}}$	$\boldsymbol{P_{21}}$	$\boldsymbol{P_{21/c}}$	$\boldsymbol{P_{212121}}$
conformation 1	4.21	2.68	2.68	1.77	2.36

conformation 2	4.84	3.09	4.93	3.52	4.36
conformation 3	2.73	0.53	2,089	0.00	1.74
conformation 4	5.54	5.31	3.30	4.80	4.46
conformation 5	5.20	3.50	3.33	2.63	4.60
conformation 6	5.70	3.87	5.79	3.56	5.32
conformation 7	6.14	9.45	11.38	7.71	8.08
conformation 8	7.62	7.58	8.44	7.48	8.66
conformation 9	7.13	6.80	6.71	7.44	5.79
conformation 10	7.63	2.34	2.67	3.00	3.12
conformation 11	5.55	3.43	3.30	3.29	4.36
conformation 12	10.14	6.06	9.14	6.50	7.88
conformation 13	10.76	5.28	9.58	8.38	12.09
conformation 14	10.25	10.12	9.46	8.13	9.81
conformation 15	11.10	6.08	9.94	3.29	10.37
conformation 16	10.95	6.06	11.86	9.86	10.18

Table S8: Relative energies (in kcal/mol) of the crystal structures of DCV-Me optimized with DFT-D.

**relative energy**	$C_{2/c}$	P_{-1}	P_{21}	$P_{21/c}$	P_{212121}
conformation 1	4.3	1.5	2.6	5.1	4.8
conformation 2	5.5	1.7	4.4	6.5	4.6
conformation 3	3.5	3.5	4.0	0.0	2.9
conformation 4	7.9	4.9	2.0	3.6	4.7
conformation 5	2.5	2.5	2.1	1.6	4.8
conformation 6	5.3	4.4	5.0	5.3	4.2
conformation 7	6.5	8.1	8.3	9.4	7.5
conformation 8	4.1	5.9	9.2	4.8	5.7
conformation 9	6.1	2.8	3.7	6.9	1.7
conformation 10	4.4	3.1	1.9	2.7	3.6
conformation 11	3.8	2.6	1.5	2.9	4.6
conformation 12	7.4	4.8	4.4	7.0	4.8
conformation 13	4.6	4.9	8.5	3.7	8.6
conformation 14	6.5	3.8	10.4	9.7	4.2
conformation 15	6.0	3.3	4.7	2.2	6.8
conformation 16	8.6	4.3	9.6	6.6	6.0

Table S9: Lattice lengths (Å) and angles (°) of the polymorph obtained by on-surface modelling (SCSP on-surface), then further optimized with FF without influence of the substrate (SCSP bulk), and the polymorph obtained by bulk modelling (BCSP) of DCV-Me.

		a	b	c	α	β	γ	Z
SCSP	*On-surface*	8.2	8.5	27.8	90.6	98.5	90.4	4

	Bulk	7.5	8.6	29.4	90.0	94.8	90.0	4
BCSP		7.5	8.3	30.6	90.0	94.2	90.0	4

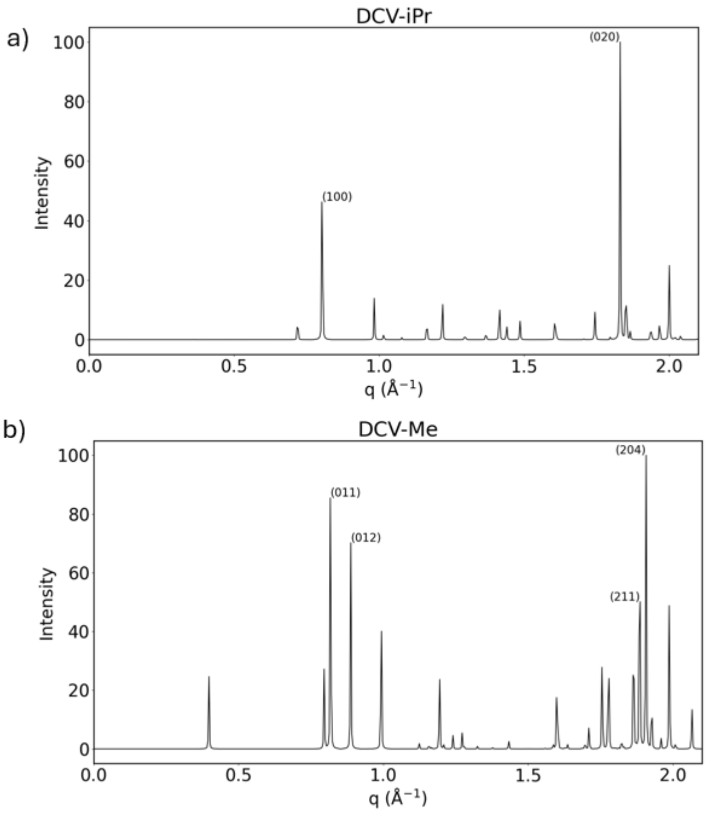

Figure S10: Simulated powder pattern of the predicted crystal structure of a) DCV-iPr and b) DCV-Me.

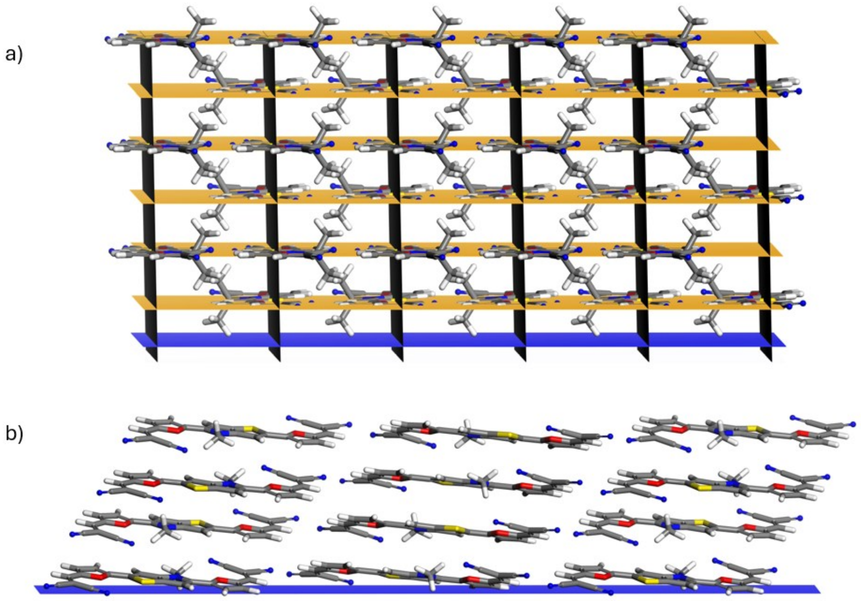

Figure S11: a) Orientation of the single crystals in the DCV-iPr film, with the (020) planes parallel to the substrate (in orange and oriented horizontally), and the (100) planes

perpendicular to the substrate (in black and oriented vertically). b) Orientation of the single crystals in the DCV-Me film, with the (204) planes parallel to the substrate.

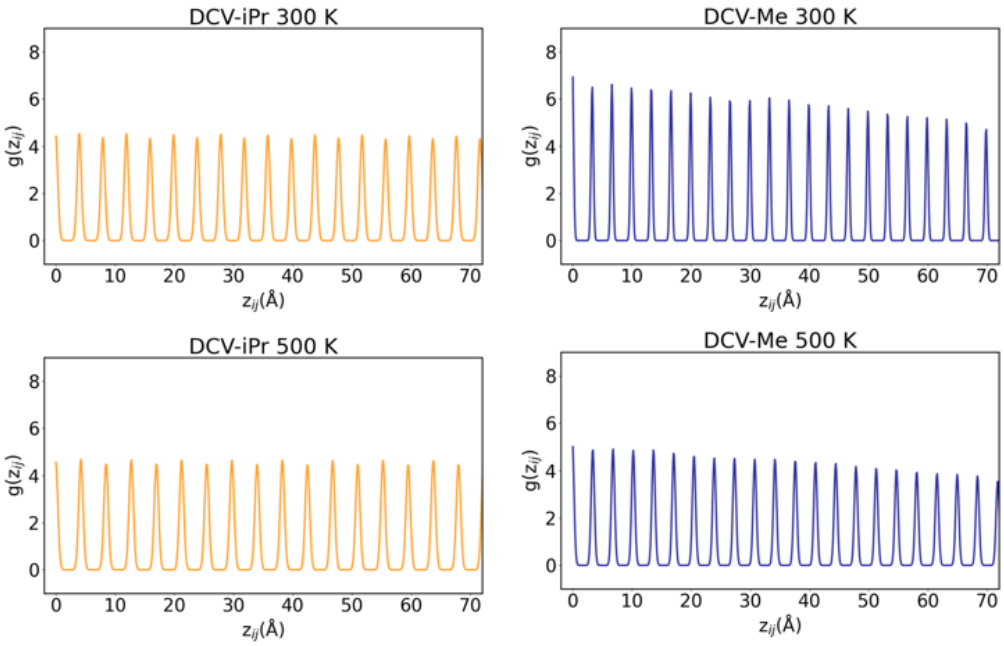

Figure S12: Distribution functions of the projection of the intermolecular centers of mass distance along the z axis (normal to the substrate and to the molecular aromatic planes) of DCV-iPr and DCV-Me supercells, obtained by replicated the SCSP unit cells (Table S6 and S9) 20 x 10 x 24 and 22 x 5 x 22 times, respectively. The functions are averaged on 200 frames of a 2 ns long dynamics performed at 300 K and 500 K. Besides the periodic peaks at the characteristic π−π stacking distance, the distributions show an important difference between the two systems, intrinsic of the different packing. In fact, while for DCV-iPr the correlation length, i.e. the distance at which the periodic peaks disappear, seems infinite or extremely large as expected from a perfectly periodic crystal, for DCV-Me the intensity of the peaks progressively fades, for an estimated correlation length of a few hundreds of nanometres. This reveals that the structure of DCV-iPr crystals is more rigid than that of DCV-Me ones, which are instead more plastic, and also that the correlation lengths measured by X-ray scattering cannot be straightforwardly compared between the two systems, since they do not depend solely on the crystallite dimensions but they are also reflecting the different dynamics of the molecules inside the two crystal lattices.

Section S8: Further details on thin-film structures

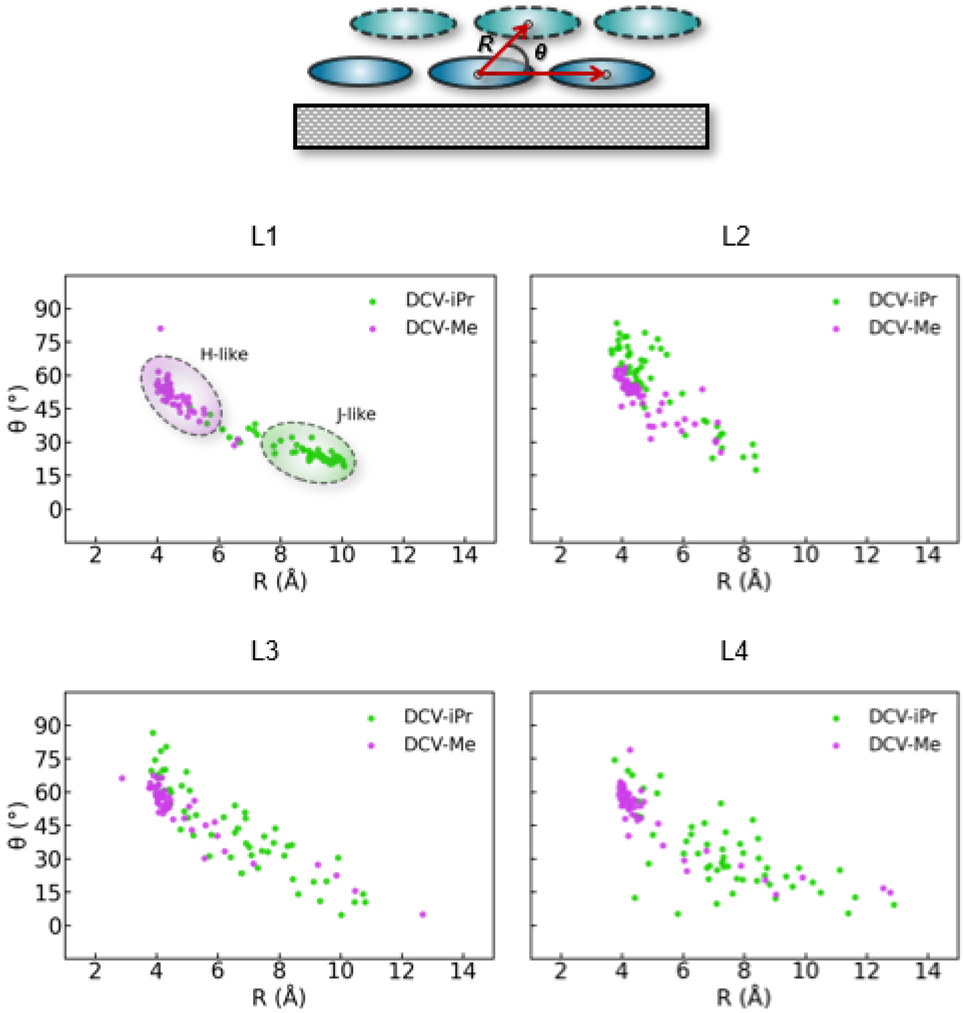

Figure S13: Correlation between the intermolecular distance R and the interlayer angle θ (see main text for their definition) for molecules in a given monolayer and their closest neighbours on the upper monolayer for DCV-Me and DCV-iPr depositions. Here the layer-partition is done considering the COM distance between molecules in the orthogonal direction at 3.5 A apart.

Section S9: Hamiltonian construction and matrix elements

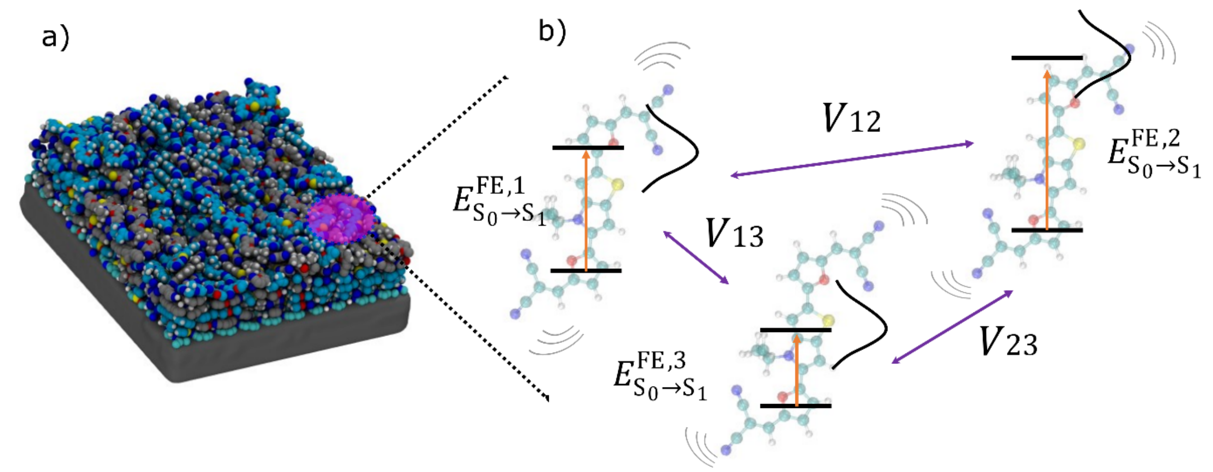

Figure S14: a) Representation of the thin-film system. b) Representation of the energetic disorder coming from electrostatic and conformational contributions.

The Hamiltonian in Eq. 1 in the main text is built for both thin-film morphologies prepared by vacuum deposition simulations as well as crystalline samples built by replicating in space the unit-cells in Table 2 of the main text. The Hamiltonian is constructed using excitation energies, couplings and electron-phonon couplings, all calculated using TDDFT. In Figures S14, we represent how the Frenkel Hamiltonian H_{FE} in Eq. 2 is constructed. The site energies in disordered morphologies are different for each molecule: $E^{(\mathrm{film},k)}_{\mathrm{S_0 \to S_1}}(\boldsymbol{R}_{\mathrm{lf}}) = E'^{(\mathrm{vac},k)}_{\mathrm{S_0 \to S_1}}(\boldsymbol{R}_{\mathrm{lf}}) + \Delta^{(\mathrm{film},k)}_{\mathrm{env}}(\boldsymbol{R}_{\mathrm{lf}}) - \lambda^{\mathrm{rel}}_{\mathrm{hf}}$ where $E'^{(\mathrm{vac},k)}_{\mathrm{S_0 \to S_1}}(\boldsymbol{R}_{\mathrm{lf}})$ is the (adiabatic) energy of the $\mathrm{S_0} \to \mathrm{S_1}$ electronic transition in solution and $\Delta^{(\mathrm{film},k)}_{\mathrm{env}}(\boldsymbol{R}_{\mathrm{lf}})$ accounts for polarization and other electrostatic effects between molecules (see main text). The off-diagonal elements of H_{FE} are the excitonic couplings, $V_{kl}(\boldsymbol{R}_{\mathrm{lf}}(t))$, which describe the interactions between tightly a bound electron-hole pair sitting either on molecules k or l and allow for excitation energy transfer between the two.

Finally, the localized excitations are coupled to the nuclear degrees of freedom Eq. 3 in the main text using appropriately computed by effective intramolecular modes tied to the formation of excitonic states on the different molecules (see Section S4).

Multi-state fragment excitation difference fragment charge difference approach

The MS-FED-FCD extends the capabilities of the previously developed diabatization schemes such as the 2-state fragment excitation difference (FED)[8] and fragment charge difference (FCD)[9,10] approaches. All these methods, which have been described in detail elsewhere[11–14], involved the partition of a given donor acceptor pair into two fragments and by using appropriate additional operators, the adiabatic Hamiltonian of the dimer (formed by two or more adiabatic states of the system) is transformed into a diabatic basis, which allows a direct evaluation of the couplings between FE states or intermolecular charge transfer (CT) states from the diabatic Hamiltonian matrix. When dealing with closely packed molecules, the

adiabatic states of the interacting pair might be partially mixed with several states of different characters (*e.g.*, FE and CT excitons). This is where the MS-FED-FCD is particularly useful and goes beyond standard FED and FCD algorithms, which only allow to diabatize two states at a time (*i.e.*, two FEs or two CTs respectively). Specifically, MS-FED-FCD allows us to include several adiabatic excited states of the donor-acceptor system in the diabatization procedure to ensure a complete de-mixing between excitations of different nature and an optimal reconstruction of the localized FE and CT states, even in cases where a given adiabatic state is the combination of many diabatic states of both donor and acceptor. The TDDFT calculations (at the ωB97X-D/6-31G(d,p) level of theory) required to obtain the excitonic couplings mentioned above were performed on a number of molecular pairs (Figures S15 and S16) extracted from crystalline samples within a 20 Å radius, with a minimum intermolecular distance of 4 Å. The diabatization procedure included 20 adiabatic excited states. The results are reported in Tables S10-13.

We note in passing that, since MS-FED-FCD allows for the calculation of couplings between Frenkel exciton (FE) and charge-transfer (CT) states, the mixing between these states could be accounted for by introducing an additional block in the Hamiltonian in Eq. 1.[15] In this case, besides the excitonic couplings V_{kl}, the coupling matrix elements responsible for exciton splitting are the photoinduced electron transfer (PET) couplings (D_{e}) and photoinduced hole transfer (PHT) couplings (D_{h}). However, the complexity of the problem increases significantly for large system sizes. Moreover, we found that CT states are typically ~1 eV above FE states at the ωB97X-D level of theory, except for a few pairs with very small intermolecular distances (see Table S12, S13), so we assume that the mixing between FE and intermolecular CT states can be disregarded. We also verified that similar findings hold for other optimally tuned LC-ωhPBE functionals with CT being higher in energy than FE states by ~0.5 eV depending on the pair considered.

Transition ESP charges

Although MS-FED-FCD is a valuable method, its high computational cost makes it impractical for evaluating long-range excitonic interactions across all molecular pairs in a supercell of large aggregates, like those studied here. An alternative, more efficient approach that has been successfully used to compute Coulombic interactions in organic crystals[14] involves transition charges obtained by fitting the electrostatic potential (TrESP). Within this framework, excitonic coupling is described as the Coulomb interaction between the TrESP charges of donor and acceptor molecules:

$$V_{kl} = \frac{1}{\varepsilon} \sum_{A \in k} \sum_{B \in l} \frac{q_A^T q_B^T}{|\boldsymbol{r}_A - \boldsymbol{r}_B|} \tag{S5}$$

where the indices A and B run over the atoms of molecules k and l, respectively, q_A^T, q_B^T are the transition charges and $\boldsymbol{r}_A$, $\boldsymbol{r}_B$ are the positions of atoms A and B, respectively. TrESP charges were obtained as proposed by Renger *et al.* in Ref[16], by fitting the electrostatic potential generated by the transition density. TrESP charges were calculated for the isolated molecule

(employing our ωB97X-D /6-31G(d,p) level of theory) and then used to compute all the long-range excitonic interactions in the Hamiltonian in Eq. 1 (even for pairs further away in space than those reported in Fig. S2). A screening factor ε, representing the dielectric constant, can be applied to include polarization effect due to the environment.

Excitonic coupling sign

We note that the excitonic coupling sign between two molecules of a given dimer extracted from the solid-state is arbitrary as it depends on the phase of the interacting molecular transition densities. However, it is vital to ensure a consistent relative phase and coupling sign when constructing the exciton Hamiltonian (Eq. 2) of a given system. The sign combination has an important impact on the electronic and optical properties[17,18]. The requirement of a consistent sign combination is straightforwardly fulfilled when using atomic TrESP charges to represent transition densities and to calculate excitonic couplings, V_{kl}. The phase of the molecular transition density is determined by construction by the order of atomic TrESP charges that is kept consistent for all the molecules in the supercell. In fact, the order of the TrESP charges depends on the specific atom ordering which is the same for all the molecules in the crystal and fulfills translational symmetry. The TrESP approach allows in principle to univocally distinguish positive (which we called H-like) vs negative (indicated with J-like) interactions computing a signed transition-dipole-corrected coupling $\tilde{V}_{kl}$ as

$$\tilde{V}_{kl} = \mathrm{sgn}(\boldsymbol{d}_k \cdot \boldsymbol{d}_l)V_{kl} \qquad \text{(S6)}$$

where the term in between parenthesis is the scalar product of the transition dipoles obtained using atomic TrESP charges as $\boldsymbol{d} = \sum_I q_I^T \boldsymbol{r}_I$ (I runs over the atomic positions of the molecule and q_I^{T} are the atomic TrESP charges). If all the dipoles are oriented in the same direction, then $\tilde{V}_{kl} = V_{kl}$. See Fig. 2 for a visual representation of H- vs J-like interactions.

Extracted dimers

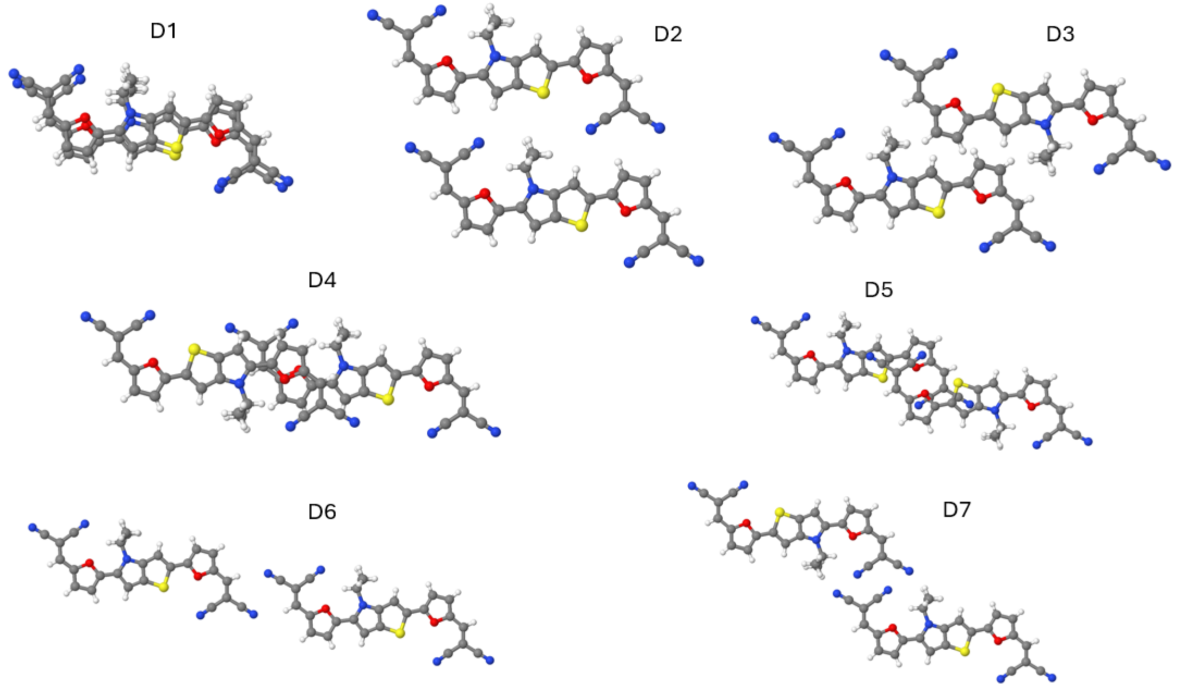

Figure S15: DCV-iPr unique dimers with a COM distance within 20 Å and a minimum distance within 4 Å. These dimers were taken from a 2x2x2 supercell obtained from the experimental unit-cell in Table 2.

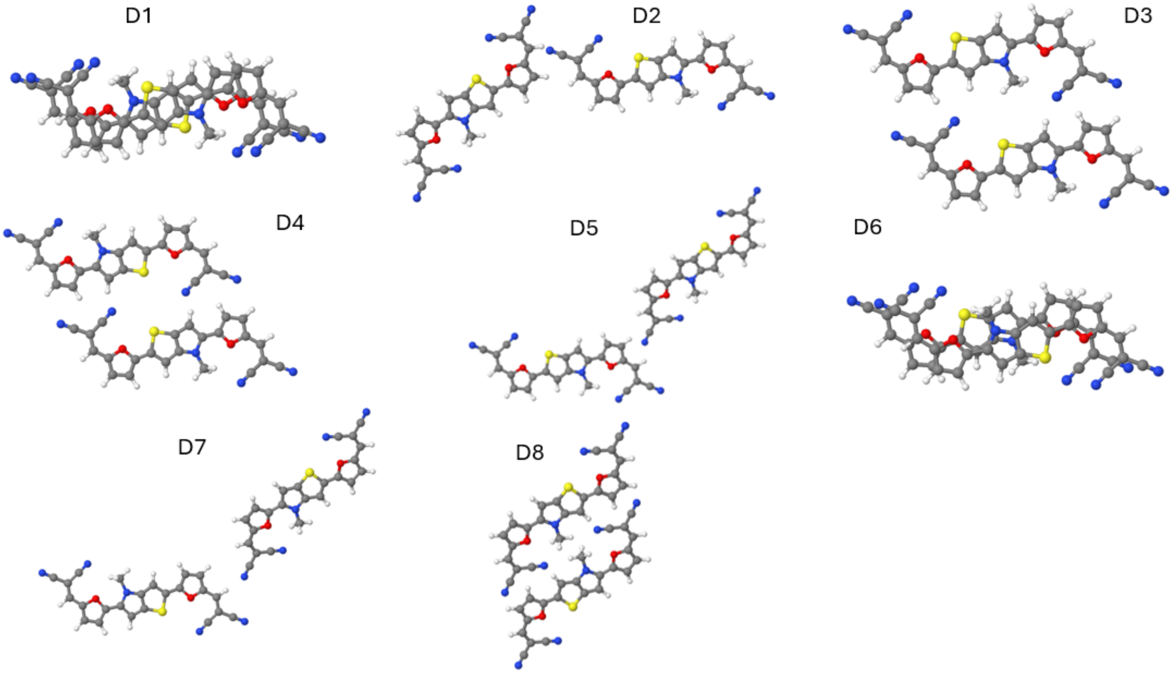

Figure S16: DCV-Me unique dimers with COM distance within 20 Å and minimum distance within 4 Å. These dimers were taken from a 2x2x2 supercell obtained from the unit-cell predicted by BCSP and refined at DFT-D level as indicated in Table 2.

Table S10: Computed excitonic couplings of the nearest neighbour pairs of DCV-iPr molecules. All couplings in meV and distances in Å. The data refers to the pairs in Fig. S15 and were obtained at ωB97X-D /6-31G(d,p) level of theory.

Dimer	COM Distance (Å)	Min. Distance (Å)	$\tilde{V}_{kl}^{\text{MS-FED-FCD}}$ [meV]	$\tilde{V}_{kl}$ [meV]
D1	6.82	2.78	76.47	75.95
D2	8.70	2.48	76.54	57.82
D3	9.28	2.44	35.52	34.73
D4	10.18	2.21	28.51	18.71
D5	10.99	3.40	-27.43	-29.64
D6	19.68	2.54	-52.55	-51.55
D7	14.98	2.78	-44.60	-43.36

Table S11: Computed excitonic couplings of the nearest neighbour pairs of DCV-Me molecules. All couplings in meV and distances in Å. The data refers to the pairs in Fig. S16 and were obtained at ωB97X-D /6-31G(d,p) level of theory.

Dimer	COM Distance (Å)	Min. Distance (Å)	$\tilde{V}_{kl}^{\text{MS-FED-FCD}}$ [meV]	$\tilde{V}_{kl}$ [meV]
D1	3.71	2.53	190.61	182.45
D2	16.42	2.85	-47.58	-45.50
D3	8.32	2.57	92.24	67.08
D4	9.06	2.52	68.10	53.77
D5	16.71	3.00	-45.48	-40.71
D6	3.78	2.86	191.44	178.75
D7	18.04	2.23	-51.05	-48.69
D8	9.20	2.57	79.47	42.43

Table S12: Computed (mean) diabatic energies of FE and intermolecular CT states, along with the corresponding PHT and PET couplings between FE and CT, given in absolute values for the DCV-iPr molecular pairs reported in Fig. S15. Energies are in eV, couplings in meV, and distances in Å. The ωB97X-D/6-31G(d,p) level of theory was used.

Dimer	COM Distance (Å)	Min. Distance (Å)	CT energy (eV)	FE energy (eV)	Offset (eV)	$\lvert D_h \rvert$ (meV)	$\lvert D_e \rvert$ (meV)
D1	6.82	2.78	3.85	2.67	1.19	0.15	0.38
D2	8.70	2.48	3.64	2.58	1.06	1.27	9.63
D3	9.28	2.44	3.85	2.68	1.17	3.59	21.78
D4	10.18	2.21	3.04	2.65	0.39	10.00	97.83
D5	10.99	3.40	3.37	2.62	0.74	36.02	53.58
D6	19.68	2.54	4.44	2.66	1.78	0.94	10.61
D7	14.98	2.78	4.17	2.65	1.52	1.84	8.74

Table S13: Computed (mean) diabatic energies of FE and intermolecular CT states, along with the corresponding PHT and PET couplings between FE and CT, given in absolute values for the DCV-Me molecular pairs reported in Fig. S16. Energies are in eV, couplings in meV, and distances in Å. The ωB97X-D/6-31G(d,p) level of theory was used.

Dimer	COM Distance (Å)	Min. Distance (Å)	CT energy (eV)	FE energy (eV)	Offset (eV)	$\|D_h\|$ (meV)	$\|D_e\|$ (meV)
D1	3.71	2.53	2.73	2.45	0.28	59.79	110.82
D2	16.42	2.85	3.98	2.46	1.52	14.27	19.15
D3	8.32	2.57	3.45	2.40	1.05	2.20	8.92
D4	9.06	2.52	3.47	2.39	1.08	2.66	4.78
D5	16.71	3.00	3.98	2.46	1.52	6.71	9.77
D6	3.78	2.86	2.76	2.46	0.30	65.50	50.23
D7	18.04	2.23	4.13	2.46	1.66	1.20	5.36
D8	9.20	2.57	3.46	2.39	1.07	1.43	2.53

Section S10: Spectral density and dynamic conformational disorder

The fluctuations associated with a time signal can be analysed using the spectral density approach. Denoting the autocorrelation function of a given time , as $C_a(t)$, we have:

$$C_a(t) = \langle \delta\Delta E(0)\delta\Delta E(t) \rangle \qquad \text{(S7)}$$

where $\delta\Delta E(t) = \delta\Delta E(t) - \langle \delta\Delta E \rangle$ and $\Delta E(t)$ the vertical energy gap. The spectral density function, $S_a(\omega)$, is obtained from the cosine transform of $C_a(t)$ as:

$$\frac{S_a(\omega)}{\omega} = \frac{\beta}{2}\int_0^{+\infty} dt \cos(\omega t) C_a(t) \qquad \text{(S8)}$$

where $\beta = 1/K_B T$, allowing $C_a(t)$ to be expressed in terms of the inverse transform as:

$$C_a(t) = \frac{4}{\beta\pi}\int_0^{+\infty} d\omega \cos(\omega t)\frac{S_a(\omega)}{\omega} \qquad \text{(S9)}$$

The autocorrelation function at $t = 0$ is equivalent to the variance of $\Delta E(t)$, $C_a(0) = \langle \delta\Delta E^2 \rangle = \sigma^2$. So, we can calculate the running integral of Eq. S12 with $t = 0$ up to a frequency ω, which corresponds to the variance of the time series including all frequency contributions up to ω. Working in terms of the root-mean-square fluctuation $\sigma(\omega)= \sqrt{C_a(0)}$, we have:

$$\sigma(\omega) = \left[\frac{4}{\beta\pi}\int_0^{+\omega} d\omega' \frac{S_a(\omega')}{\omega'}\right]^{1/2} \qquad \text{(S10)}$$

one can link the spectral density obtained from an MD at finite temperature to the relaxation energy calculated with Eq. S3. In linear response, one can prove that:

$$\lambda^{\text{rel}} = \frac{\beta}{2}\langle \delta\Delta E^2 \rangle = \frac{2}{\pi}\int_0^{+\omega} d\omega' \frac{S_a(\omega')}{\omega'} \quad \text{(S11)}$$

Table S14: Relaxation energy (λ_i^{rel}) obtained from normal mode analysis (NMA) for DCV-iPr molecule and e cosine transform of the excitation energies computed for molecules extracted from the top layer (L6) or extracted from the lower layer (L1).

	NMA	SPD (L1)	SPD (L6)
λ^{rel}	0.164	0.141	0.135
$\sigma^{\text{tot.}}$	-	0.088	0.083
$\lambda_{\text{lf}}^{\text{rel}}$	0.041	0.041	0.050
$\sigma^{\text{hom.}}$	0.046	0.047	0.051

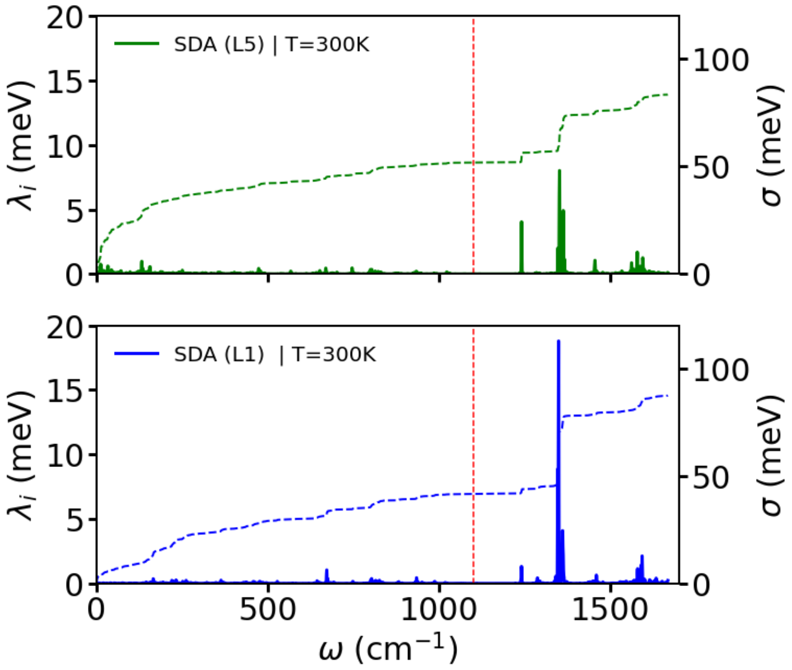

Figure S17: Relaxation energy (λ_i^{rel}) obtained from the cosine transform of the excitation energies computed at TDDFT level, by sampling every 10 fs a 20 ps long MD run at 300 K. Top panel the molecule followed along dynamics is extracted from the top layer, bottom panel the molecule followed along dynamics is extracted from the lower layer (L1).

Section S11: Further details on calculated solid-state spectra

The normalized absorption spectrum is calculated by using[19,20] Eq S3, using the ground state wavefunction $\Psi^{(g)}$ and the wavefunction of a given eigenstate $\Psi^{(j)}$ of the system obtained diagonalizing the Hamiltonian in Eq. 1.
The emission spectrum is calculated analogously by using[19,20]:

$$S(\omega) = \sum_j \frac{e^{-(\omega_j-\omega_{j=0})/k_BT}}{\sum_j e^{-(\omega_j-\omega_{j=0})/k_BT}} \sum_{v_i=0,1,2} \left(\omega_j - v_i\omega_{\mathrm{eff}}\right)^3 I^{v_i} W\left(\omega - \omega_j + v_i\omega_{\mathrm{eff}}\right) \qquad \text{(S12)}$$

where the first sum runs over the adiabatic excited states (j) of the system that are Boltzmann averaged to consider the temperature effect in emission (which otherwise would take place only from the lowest energy excited state). The emission terminates on the ground electronic state creating a vibronic progression with peak frequencies at $\omega_j + v_i\omega_{\mathrm{eff}}$ (where v_i is the number of total vibrational quanta). I^{v_i} represents the intensity of the peaks whose explicit mathematical expressions are given in Ref.[19].

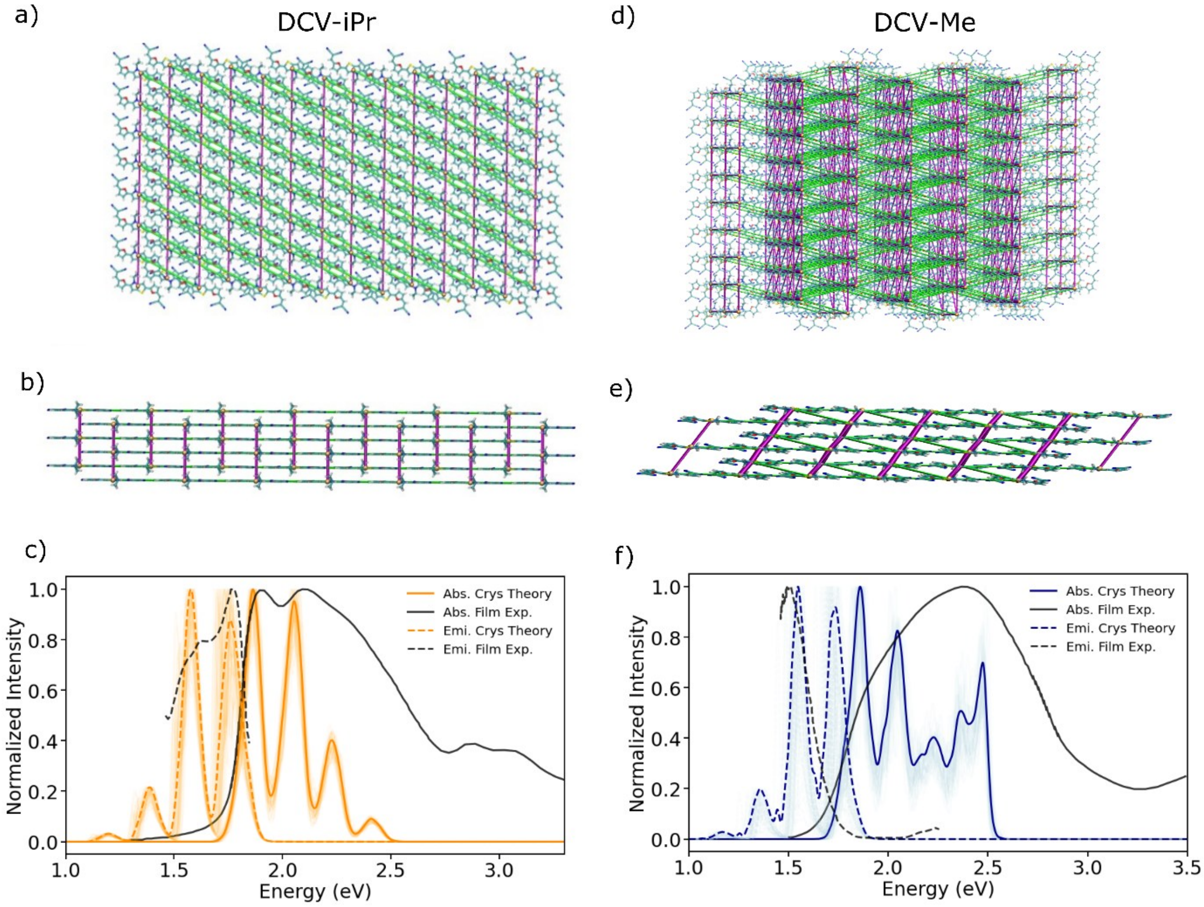

Figure S18: Excitonic coupling networks for DCV-iPr and DCV-Me crystals, represented from a top view in (a) and (d), respectively, and a lateral view in (b) and (e). H-like (J-like)

interactions are shown as green (magenta) segments. The supercells used for constructing the Hamiltonian were 8×3×7 (336 mol in total) for DCV-iPr (replicated using the experimentally predicted structure) and 9×3×3 (324 mol in total) for DCV-Me (replicated using the BSCP structure). The computed spectra shown in (c) and f) for DCV-iPr and DCV-Me, respectively, were shifted by -0.38 eV.

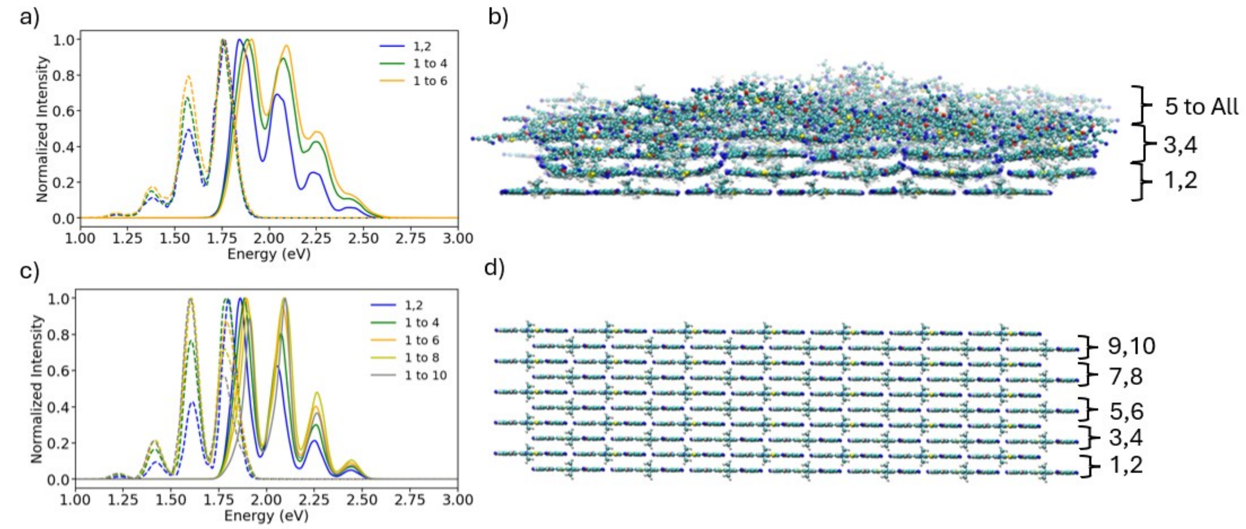

Figure S19: Simulated spectra of DCV-iPr as a function of layer growth for the vacuum deposited thin-film structure panel a) and crystalline structure c). Layer partitioning is shown in panels b and d for both morphologies (see also Fig. 3c for vacuum deposited structure). All computed spectra are shifted by -0.38 eV.

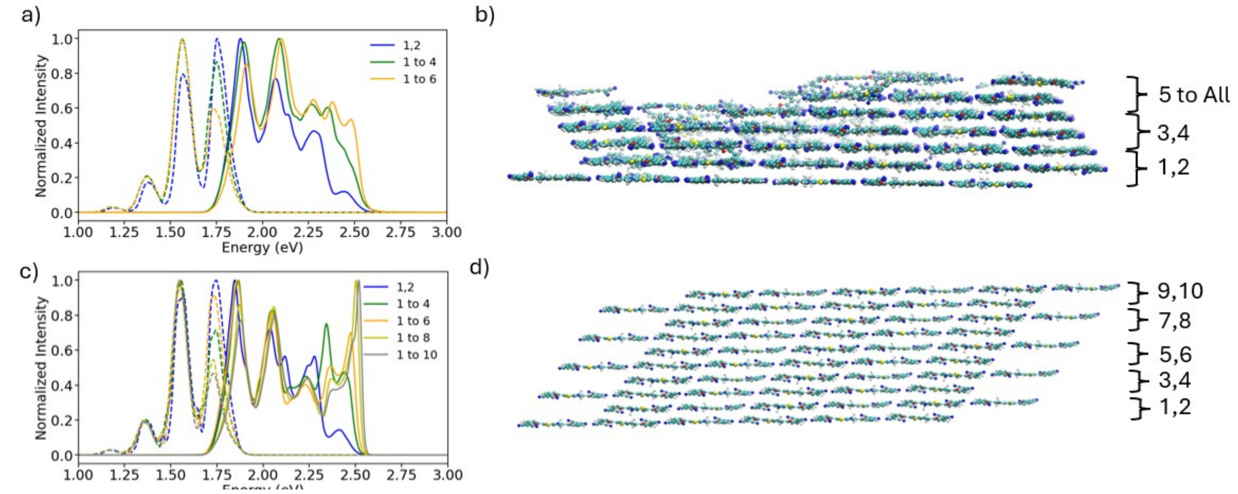

Figure S20: Simulated spectra of DCV-Me as a function of layer growth for the vacuum deposited thin-film structure panel a) and crystalline structure c). Layer partitioning is shown in panels b and d for both morphologies (see also Fig. 3c for vacuum deposited structure). All computed spectra are shifted by -0.38 eV.

Section S12: Further experimental optical data

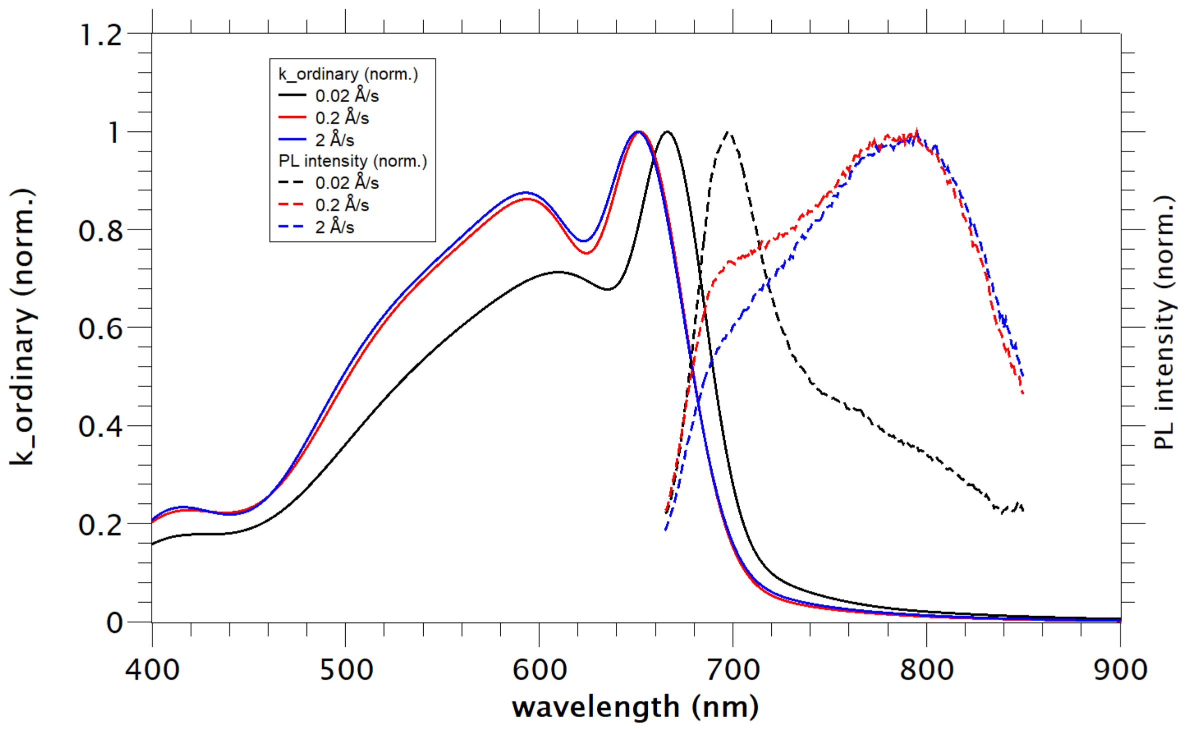

Figure S21: normalized extinction coefficient $k_{ordinary}$ and normalized PL intensity spectrum for a series of DCV-iPr layers deposited at different evaporation rates onto silicon wafers with 500nm thick oxide layers followed by a 10nm thick layer of fullerene C_{60}. $k_{ordinary}$ was determined by an anisotropic fit to ellipsometry data. The C_{60} interlayer was used here to provide the same seed layer as in the solar cell structure. GIWAXs analysis indicates that all layers preferentially grow face on with vertical crystallite sizes of at least 8nm for all studied growth rates. Moreover, the peak positions in both 1D and 2D GIWAXs are independent on growth rate and largely agree with the bulk crystalline structure. However, the orientational disorder increases with decreasing rate leading to less optical anisotropy while AFM micrographs indicate higher surface roughness and larger grains. While it is plausible to assume that slower growth should lead to better crystalline order, we cannot give an unambiguous proof for this assumption from the morphology analysis. In any case, layers grown at low rate feature remarkably dominant 0-0-transitions in both absorption and PL. From the comparison with simulated spectra for different degrees of order, we may conclude that the layers grown at low rate exhibit a higher degree of short-range crystalline order.

Section S13: PHJ-type devices

PHJ-type devices based on the absorber BODIPY-Pyr-Cl_2 have been prepared with the following layer sequence: glass / ITO / C60:EDN003 (5 nm, 5:1) / C60 (10 nm) / BODIPY-Pyr-Cl_2 (8 -12 nm) / TaTm (5 nm) / TaTm:NDP-9 (30 nm) / MoOx (20 nm) / Al (100 nm) where EDN003 is an n-dopant delivered by Novaled GmbH. The samples have been encapsulated and measured with the same LED based sun simulator that was used for the BHJ+

devices. The continuous increase of j_{sc} with the thickness of the absorber indicates that excitons generated in BODIPY-Pyr-Cl_2 have a diffusion length of at least 12 nm so that they can reach the interface to C_{60} where they are efficiently separated into carrier pairs.

Table S15: Photovoltaic parameter of PHJ-type device based on BODIPY-Pyr-Cl_2 with 8, 10, and 12 nm, respectively

PHJ thickness	J_{sc} (mA/cm^2)	V_{oc} (V)	FF (%)	PCE (%)
8nm	7.2	0.97	77.0	5.3
10nm	7.9	0.97	77.3	6.0
12nm	8.7	0.98	77.2	6.6

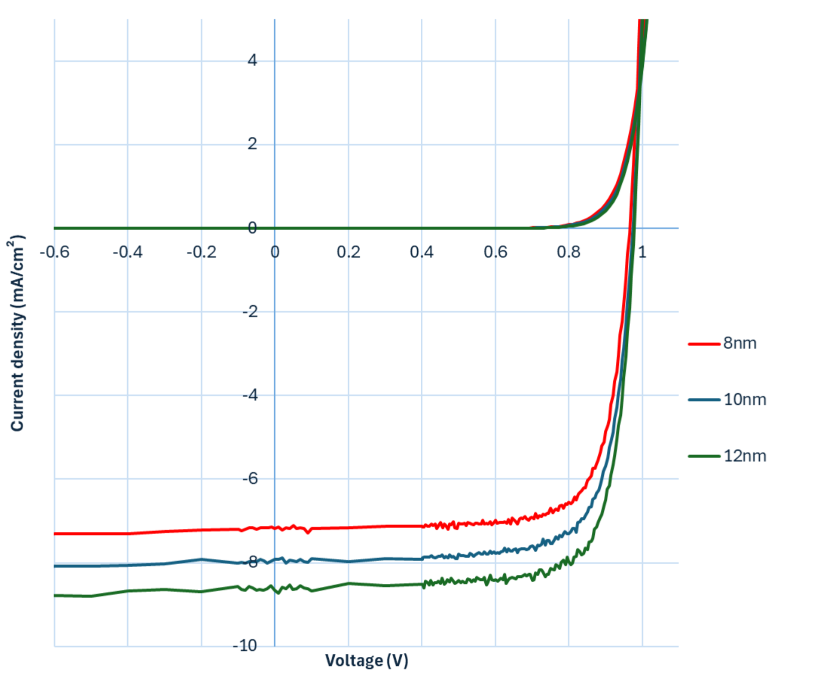

Fig. S22: J-V-characteristics measured with mask for 8, 10, and 12 nm BODIPY-Pyr-Cl_2 active layer thickness in a PHJ device.

Section S14: Steepness of the absorption edge

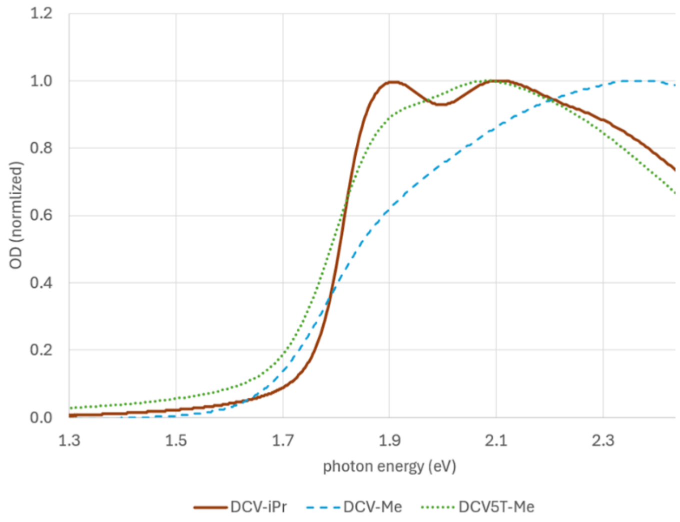

Fig. S23: Normalized optical density spectra of 30nm thick vacuum deposited films of DCV-iPr, DCV-Me and DCV5T-Me [21] plotted versus photon energy. Adapted from Ref. [21] Copyright © 2012 American Chemical Society.

Section S15: Estimation of the apparent Urbach energy

An estimation for the apparent Urbach energy and steepness at room temperature can be calculated for DCV-iPr:C_{60} from Fig. 6a. The following values are extracted: $E_{U,app}$ = 47 meV, steepness of the absorption tail = 21 eV^{-1}. Please note, that the fit window is small due to the influence of the CT-transition on the subgap EQE. We consider an error range of ± 2 meV (± 1 eV^{-1}) for apparent Urbach energy and steepness of the absorption tail.

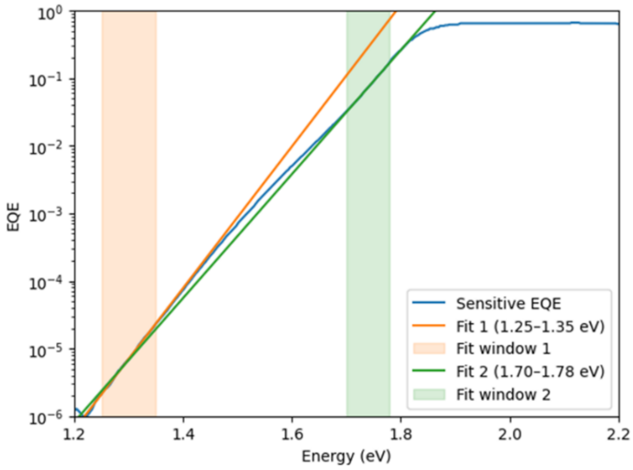

Fig. S24: Fit of the apparent Urbach energy of DCV-iPr:C_{60} (Fit window 2, green line) and the fit to the tail of CT absorption shoulder (fit window 1, orange line) using sensitive EQE data from Fig. 6a.

However, in this spectral region, the influences of the CT state and of energetic disorder are difficult to disentangle. Therefore, a fit to the tail of CT absorption shoulder in the range of 1.25-1.35 eV is more directly related to energetic disorder. Here, we find an Urbach energy of $E_{U,CT} = (41.6 \pm 0.8)$ meV. While the apparent Urbach energy is comparable to reported Urbach energies of commercial a-Si:H thin film solar cells ($E_U \approx 47$ meV)[22] and solution-processed PM6:Y25 devices ($E_U = 48.5$ meV) [23], the Urbach energy of the CT shoulder is significantly smaller. On the other hand, a tail energy above k_BT is certainly not ideal for efficient solar cells and indicates harmful influence of both intermolecular and intramolecular disorder which negatively affects both V_{oc} and carrier mobility and thus FF.

References

[1] J. Tomasi, B. Mennucci, R. Cammi, "Quantum Mechanical Continuum Solvation Models," *Chem. Rev.* **2005**, *105*, 2999–3094.

[2] V. Coropceanu, J. Cornil, D. Silva, D. A, Y. Olivier, R. Silbey, J. L. Bredas, D. A. da Silva Filho, J.-L. J.-L. Brédas, Y. Olivier, R. Silbey, J.-L. J.-L. Brédas, "Charge Transport in Organic Semiconductors.," *Chem. Rev.* **2007**, *107*, 926–52.

[3] O. G. Ziogos, S. Giannini, M. Ellis, J. Blumberger, T. Y. Centre, "Supporting Information for Identifying High-Mobility Tetracene Derivatives Using a Non-Adiabatic Molecular Dynamics Approach," **2019**, 1–22.

[4] H. Oberhofer, K. Reuter, J. Blumberger, "Charge Transport in Molecular Materials: An Assessment of Computational Methods," *Chem. Rev.* **2017**, *117*, 10319–10357.

[5] J. Cerezo, F. Santoro, "FCclasses3 : Vibrationally-resolved Spectra Simulated at the Edge of the Harmonic Approximation," *J. Comput. Chem.* **2023**, *44*, 626–643.

[6] F. J. Avila Ferrer, F. Santoro, "Comparison of Vertical and Adiabatic Harmonic Approaches for the Calculation of the Vibrational Structure of Electronic Spectra," *Phys. Chem. Chem. Phys.* **2012**, *14*, 13549.

[7] J. Aragó, A. Troisi, "Regimes of Exciton Transport in Molecular Crystals in the Presence of Dynamic Disorder," *Adv. Funct. Mater.* **2016**, *26*, 2316–2325.

[8] C.-P. Hsu, Z.-Q. You, H.-C. Chen, "Characterization of the Short-Range Couplings in Excitation Energy Transfer," *J. Phys. Chem. C* **2008**, *112*, 1204–1212.

[9] A. A. Voityuk, "Estimation of Electronic Coupling in π -Stacked Donor-Bridge-Acceptor Systems: Correction of the Two-State Model," *J. Chem. Phys.* **2006**, *124*, 1–7.

[10] C.-H. Yang, C.-P. Hsu, "A Multi-State Fragment Charge Difference Approach for Diabatic States in Electron Transfer: Extension and Automation," *J. Chem. Phys.* **2013**, *139*, 154104.

[11] L. Cupellini, M. Corbella, B. Mennucci, C. Curutchet, "Electronic Energy Transfer in Biomacromolecules," *WIREs Comput. Mol. Sci.* **2019**, *9*, 1–23.

[12] M. Nottoli, S. Jurinovich, L. Cupellini, A. T. Gardiner, R. Cogdell, B. Mennucci, "The Role of Charge-Transfer States in the Spectral Tuning of Antenna Complexes of Purple Bacteria," *Photosynth. Res.* **2018**, *137*, 215–226.

[13] J. Tölle, L. Cupellini, B. Mennucci, J. Neugebauer, "Electronic Couplings for Photo-Induced Processes from Subsystem Time-Dependent Density-Functional Theory: The Role of the Diabatization," *J. Chem. Phys.* **2020**, *153*, 184113.

[14] S. Giannini, W.-T. Peng, L. Cupellini, D. Padula, A. Carof, J. Blumberger, "Exciton Transport in Molecular Organic Semiconductors Boosted by Transient Quantum Delocalization," *Nat. Commun.* **2022**, *13*, 2755.

[15] S. Giannini, D. J. C. Sowood, J. Cerdá, S. Frederix, J. Grüne, G. Londi, T. Marsh, P. Ghosh, I. Duchemin, N. C. Greenham, K. Vandewal, G. D'Avino, A. J. Gillett, D. Beljonne, "On the Role of Charge Transfer Excitations in Non-Fullerene Acceptors for Organic Photovoltaics," *Mater. Today* **2024**, *80*, 308–326.

[16] M. E. Madjet, A. Abdurahman, T. Renger, "Intermolecular Coulomb Couplings from Ab Initio Electrostatic Potentials: Application to Optical Transitions of Strongly Coupled Pigments in Photosynthetic Antennae and Reaction Centers," *J. Phys. Chem. B* **2006**, *110*, 17268–17281.

[17] N. J. Hestand, H. Yamagata, B. Xu, D. Sun, Y. Zhong, A. R. Harutyunyan, G. Chen, H.-L. Dai, Y. Rao, F. C. Spano, "Polarized Absorption in Crystalline Pentacene: Theory vs Experiment," *J. Phys. Chem. C* **2015**, *119*, 22137–22147.

[18] N. J. Hestand, F. C. Spano, "Molecular Aggregate Photophysics beyond the Kasha Model: Novel Design Principles for Organic Materials," *Acc. Chem. Res.* **2017**, *50*, 341–350.

[19] F. C. Spano, "Absorption and Emission in Oligo-Phenylene Vinylene Nanoaggregates: The Role of Disorder and Structural Defects," *J. Chem. Phys.* **2002**, *116*, 5877–5891.

[20] N. J. Hestand, F. C. Spano, "Expanded Theory of H- and J-Molecular Aggregates: The Effects of Vibronic Coupling and Intermolecular Charge Transfer," *Chem. Rev.* **2018**, *118*, 7069–7163.

[21] R. Fitzner, E. Mena-Osteritz, A. Mishra, G. Schulz, E. Reinold, M. Weil, C. Körner, H. Ziehlke, C. Elschner, K. Leo, M. Riede, M. Pfeiffer, C. Uhrich, P. Bäuerle, "Correlation of π-Conjugated Oligomer Structure with Film Morphology and Organic Solar Cell Performance," *J. Am. Chem. Soc.* **2012**, *134*, 11064–11067.

[22] C. Kaiser, O. J. Sandberg, N. Zarrabi, W. Li, P. Meredith, A. Armin "A universal Urbach rule for disordered organic semiconductors" *Nat. Commun.* **2021**, *12*, 3988. DOI: 10.1038/s41467-021-24202-9

[23] C. Zhang, A. Mahadevan, J. Yuan, J. K. W. Ho, Y. Gao, W. Liu, H. Zhong, H. Yan, Y. Zou, S.-W. Tsang, S. K. So "Unraveling Urbach Tail Effects in High-Performance Organic Photovoltaics: Dynamic vs Static Disorder" *ACS Energy Lett.* **2022**, *7*, 1971-1979. DOI: 10.1021/acsenergylett.2c00816
